# Structure and activation mechanism of the hexameric plasma membrane H^+^-ATPase

**DOI:** 10.1038/s41467-021-26782-y

**Published:** 2021-11-08

**Authors:** Peng Zhao, Chaoran Zhao, Dandan Chen, Caihong Yun, Huilin Li, Lin Bai

**Affiliations:** 1 Department of Biochemistry and Biophysics, School of Basic Medical Sciences, Peking University, Beijing, China; 2 Department of Structural Biology, Van Andel Institute, Grand Rapids, MI USA

**Keywords:** Cryoelectron microscopy, Fungal physiology

## Abstract

The *S. cerevisiae* plasma membrane H^+^-ATPase, Pma1, is a P3A-type ATPase and the primary protein component of the membrane compartment of Pma1 (MCP). Like other plasma membrane H^+^-ATPases, Pma1 assembles and functions as a hexamer, a property unique to this subfamily among the larger family of P-type ATPases. It has been unclear how Pma1 organizes the yeast membrane into MCP microdomains, or why it is that Pma1 needs to assemble into a hexamer to establish the membrane electrochemical proton gradient. Here we report a high-resolution cryo-EM study of native Pma1 hexamers embedded in endogenous lipids. Remarkably, we found that the Pma1 hexamer encircles a liquid-crystalline membrane domain composed of 57 ordered lipid molecules. The Pma1-encircled lipid patch structure likely serves as the building block of the MCP. At pH 7.4, the carboxyl-terminal regulatory α-helix binds to the phosphorylation domains of two neighboring Pma1 subunits, locking the hexamer in the autoinhibited state. The regulatory helix becomes disordered at lower pH, leading to activation of the Pma1 hexamer. The activation process is accompanied by a 6.7 Å downward shift and a 40° rotation of transmembrane helices 1 and 2 that line the proton translocation path. The conformational changes have enabled us to propose a detailed mechanism for ATP-hydrolysis-driven proton pumping across the plasma membrane. Our structures will facilitate the development of antifungal drugs that target this essential protein.

## Abstract (web summary)

The plasma membrane H^+^ -ATPase is responsible for maintenance of the plasma membrane potential, which provides energy for the transport of nutrients, and the plasma membrane H^+^ -ATPase in *S. cerevisiae* (Pma1) is a P3A-type ATPase that assembles and functions as a hexamer. Here, the authors present the cryo-EM structures of autoinhibited and activated native Pma1 hexamers purified with endogenous lipids and they propose a mechanism for proton pumping across the membrane by this family of H^+^ -ATPases.

## Introduction

In eukaryotes, the plasma membrane potential provides energy for the transport of nutrients and is critical for cellular viability^1^. Maintenance of the plasma membrane potential relies on the Na^+^,K^+^-ATPase in animals and the plasma membrane H^+^-ATPase in plants and fungi^2,3^. The Na^+^,K^+^-ATPases belong to the P2C subfamily of P-type ATPases, a family of ATP-driven ion pumps. The Na^+^,K^+^-ATPase uses energy from ATP hydrolysis to create and maintain an electrochemical proton gradient across the plasma membrane by pumping out three sodium ions for every two potassium ions pumped into the cell^4–7^. The plasma membrane H^+^-ATPases belongs to the P3A subfamily of P-type ATPases, which harness energy from ATP hydrolysis to extrude protons from the cell, thus establishing the membrane electrochemical proton gradient and stabilizing the cytosolic pH^4–6,8^.

The plasma membrane of *Saccharomyces cerevisiae* contains several types of microdomains that underlie numerous physiological functions, including the membrane compartment containing the arginine permease Can1 (MCC) and the membrane compartment containing the H^+^-ATPase Pma1 (MCP)^9,10^. Despite extensive studies, the precise structure and the underlying mechanism of assembly of these microdomains are not well understood. The *S. cerevisiae pma1* is an essential gene^3^. Pma1 is the most abundant protein in the yeast plasma membrane and consumes the most cellular ATP^3^. Pma1 is strictly localized to the sphingolipid-rich MCP microdomains^11–14^. Long-chain sphingolipids are necessary for Pma1 sorting and directly associated with Pma1, but the underlying molecular mechanism for this has been unknown^15,16^. It has been postulated that the membrane potential established by Pma1 may drive lateral segregation of lipids, leading to the organization of these microdomains in the plasma membrane^17^.

Like all other P-type ATPases, the plasma membrane H^+^-ATPase has a transmembrane domain (TMD) composed of ten transmembrane α-helices (TMs), an actuator domain (A-domain), a nucleotide-binding domain (N-domain), and a phosphorylation domain (P-domain)^4,5,18^. The C-terminal peptide of the plasma membrane H^+^-ATPase was reported to inhibit the enzyme activity^19–22^. Glucose activation of yeast Pma1 involves phosphorylation of this C-terminal regulatory peptide^23–25^. The plant plasma membrane H^+^-ATPases are also activated by phosphorylation of their regulatory C-terminal domains^26^.

The P-type ATPases are generally monomeric except for the plasma membrane H^+^-ATPases that assemble and function as hexamers^27–32^. A cross-linking mass spectrometric study showed that the C-terminal regulatory domain of the *Arabidopsis thaliana* plasma membrane H^+^-ATPase (AHA2) mediates the subunit-subunit interactions in the hexamer^33^. The crystal structure of C-terminal regulatory domain-truncated AHA2 is monomeric, but it provided the first molecular insights into the domain architecture and proton transport path^18,34^. The hexamer architecture and the nanometer-resolution structures of the fungi plasma membrane H^+^-ATPase were reported nearly two decades ago^27–31^. During the preparation of this manuscript, a 3.3 Å resolution cryo-EM structure of the *Neurospora crassa* Pma1 was posted in BioRxiv^35^. Despite such progress, how the yeast Pma1 interacts with membrane lipids to organize the MCP microdomains and how Pma1 switches between the autoinhibited state and the active state remain unknown. In this work, we report a high-resolution cryo-EM study of autoinhibited and activated native Pma1 hexamers embedded in endogenous lipids, which sheds light on these long-standing questions.

## Results

### Isolation and structure determination of Pma1

We tagged Pma1 with a C-terminal triple FLAG epitope in yeast cells. The endogenous tagged Pma1 was extracted from the yeast membrane with the mild detergent DDM and purified via an anti-FLAG (M2) affinity column, followed by size-exclusion chromatography (SEC) (Supplementary Fig. 1a, b). The gel filtration peak contained hexameric Pma1, as indicated by the elution volume and demonstrated by a native PAGE gel (Supplementary Fig. 1c). We found that the ATPase activity of purified Pma1 was inhibited at pH 7.4 but became active at pH 6.0 (Supplementary Fig. 1d), in agreement with previous reports^36^. Cryo-EM 2D averages showed that Pma1 maintained the hexamer architecture at both pH values (7.4 and 6.0) (Supplementary Fig. 1e, f).

**Fig. 1:**
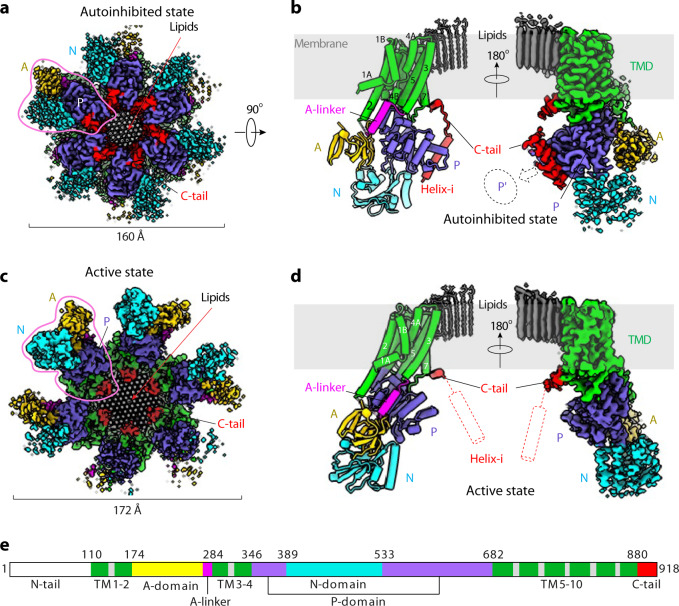
Cryo-EM structures of the autoinhibited and activated Pma1 hexamer. **a** Cryo-EM map of the Pma1 in the autoinhibited state in top view. The major domains and motifs are labeled in different colors. One subunit was highlighted by a pink shape. **b** Atomic model and cryo-EM map of the highlighted subunit of Pma1 in **a**. **c**, **d** Cryo-EM map and atomic model of the low-pH active Pma1 in the same views as the autoinhibited Pma1 in the first row (**a**, **b**). The disordered C-terminal regulatory helix-i is shown as a red cylinder in (**d**). **e** The Pma1 domain map.

We determined the cryo-EM structure of autoinhibited Pma1 hexamer at pH 7.4 to 3.2 Å resolution without imposing any symmetry; we term this structure Pma1-pH7-C1 (Fig. 1a). We found that the TMD and P-domains are well ordered and symmetric, while the A-and N-domains are asymmetric and flexible. By imposing the 6-fold symmetry, the resolution of the well-ordered core region of the hexamer was improved to 2.9 Å resolution (Pma1-pH7-C6). These maps enabled us to build the atomic model of the Pma1 hexamer (Fig. 1a, b, Supplementary Table 1, and Supplementary Figs. 2, 3).

BeF_3_^−^ is an inhibitor of P-type ATPases and stabilizes the intermediate E2P state^4^. Cryo-EM structures of Pma1 determined at pH 7.4 in the presence of 2 mM BeF_3_^−^ (Pma1-pH7-BeF) were similar to the Pma1-pH7-C1 structure in the absence of BeF_3_^−^ (Supplementary Fig. 4), indicating that BeF_3_^−^ alone is unable to convert the autoinhibited Pma1 to the active E2P state at pH 7.4. We next determined three Pma1 structures at pH 6.0: one structure in the absence of BeF_3_^−^ (Pma1-pH6) and two in the presence of 2 mM BeF_3_^−^ (Pma1-pH6-BeF-Conf1 and Pma1-pH6-BeF-Conf2). The two BeF_3_^−^ bound structures were derived from the same dataset, each accounting for 20 and 37% of the particle population, respectively (Supplementary Fig. 5–6). Interestingly, the Pma1-pH6 and Pma1-pH6-BeF-Conf2 structures are nearly identical to the autoinhibited Pma1-pH7-C1 structure, while Pma1-pH6-BeF-Conf1 is in the active E2P state (Fig. 1c, d). The EM density of BeF_3_^−^ was clearly observed in the Pma1-pH6-BeF-Conf1 3D map (Supplementary Fig. 7). This observation suggests that the autoinhibited conformation is partially stable even at pH 6.0, and that stabilization in the activated E2P conformation in vitro requires both low pH and BeF_3_^−^. To investigate if high salt facilitates Pma1 activation, we performed the ATPase activity of Pma1 in high salt at pH 7.4 and 6.0, and found Pma1 showed similar activity as in low salt (Supplementary Fig. 8). We further determined the Pma1 structure at pH 6.0 and in 500 mM NaCl (Supplementary Fig. 4). This structure is nearly identical to the autoinhibited Pma1-pH7-C6 structure, suggesting that increasing ionic strength does not help with the activation of Pma1.

### The Pma1 structure

Both the autoinhibited and activated hexameric Pma1 structures form a similar ring architecture that is 115 Å high, contains a tightly packed TMD region, and a partially flexible cytosolic region (Fig. 1a–d). As a result of the outward movement of the cytosolic region, the activated Pma1 hexamer is wider, 172 Å as compared to 160 Å in diameter in the autoinhibited hexamer. In both hexameric Pma1 structures, most transmembrane helices (TMHs) and the cytosolic P-domains of all six subunits are well ordered and arranged in perfect C6 symmetry. In contrast, most A- and N-domains are flexible, as indicated by their weaker EM densities. In fact, only one subunit in the autoinhibited state and two subunits in the activated state had good densities for rigid body fitting of the A-and N-domain, allowing modeling of the complete structure (Fig. 1a–e).

The N-terminal peptide (1–110) of the 918-residue protein is responsible for binding the A-domain but is disordered in our structure. Among the ten TMHs of each Pma1 subunit, three (TMH3, 5, and 7) line the interior surface of the hexamer ring, and four (TMH1, 2, 6, and 9) face the outside surface. The A-domain is inserted between TMH2 and TMH3 and is connected to TMH3 via a helical linker (A-linker). The P-domain and N-domain are both inserted between TMH4 and TMH5. The C-terminal peptide (880–918, termed C-tail) forms two α-helices in the autoinhibited structure of Pma1. The first C-tail helix is short, horizontal, and partially embedded in the membrane. The second C-tail helix is longer and mediates the interaction between the P-domain and the P-domain of the neighboring subunit. We have referred to this long helix as helix-i because it is ordered only in the inhibited (i) state and is disordered in the activated state, to be discussed below. In the activated Pma1 structure, BeF_3_^−^ is clearly resolved next to the phosphorylation residue D378. Remarkably, the dephosphorylation motif, the 229-TGES-232 loop in the A-domain, points towards D378, indicating that the structure is poised to dephosphorylate D378, and that the activated conformation is a transient state less stable than the autoinhibited conformation.

### An ordered membrane microdomain inside the Pma1 hexamer

**Fig. 2:**
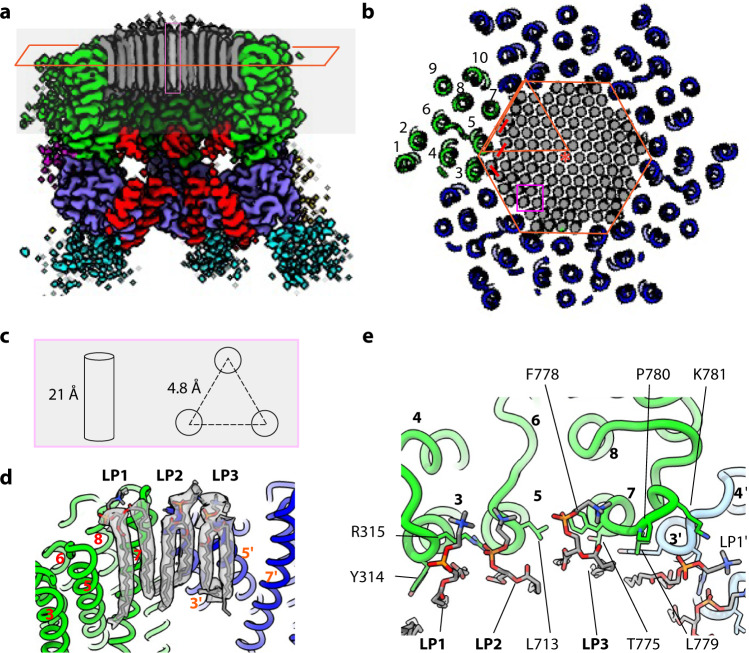
Lipids in the central hole of the Pma1 hexamer. **a** Cut-in view parallel to the membrane shows the map of autoinhibited Pma1. The rod-like lipid densities are shown in gray. A single lipid tail density is highlighted by a magenta rectangle. **b** Cut-in view from exoplasmic side of the autoinhibited Pma1. The Pma1 hexamer is shown as a cartoon, and lipids are shown as densities. The orange hexagon indicates that the lipid densities are in 6-fold symmetry, and one copy was highlighted by a triangle. The center density is highlighted by a star. Three lipids interacting with one Pma1 protomer are highlighted by three red lines. Three neighboring densities are highlighted by a magenta rectangle. **c** Length and spacing distance of the rod-like lipid tail densities. **d** Cryo-EM densities of three lipids located next to Pma1. **e** Interactions between three lipids (LP1, LP2, and LP3) and Pma1. Key residues and lipids are shown as sticks.

There are 115 well-resolved rod-like densities in the central hole of the Pma1 hexamer, at a position corresponding to the outer leaflet of the plasma membrane (Figs. 1a–d, 2a–e, Supplementary Fig. 9a). These densities were present in the previously published low-resolution EM maps of H^+^-ATPases, although it was unclear if the densities were from detergents or lipids, and whether the lipids were ordered or not^29,31^. The rod densities are packed into a hexagonal lattice (Fig. 2b). Each rod is ~21 Å tall and resembles the hydrophobic lipid tails (Fig. 2c). The rods are ~4.8 Å away from each other, consistent with a crystalline lipid monolayer. Because no lipid was added to the sample, these must be endogenous lipids copurified with the protein. The 115 rod densities suggest that the lipid microdomain is composed of 57 lipids and a single randomly distributed cavity that enables the diffusion of these lipids within the Pma1 hexamer interior. However, it is also possible that there are single-tailed lysolipids in the microdomain.

The head groups of most lipid molecules were disordered in our structure. However, each Pma1 subunit extensively interacts with three phospholipids (LP1–3) (Fig. 2d, e), and the Pma1-interacting lipids, 18 in total that line the interior surface of the Pma1 hexamer, all had well-defined densities for their head groups. Interestingly, all lipid tails in the interior of the lipid microdomain are straight and likely saturated acyl chains. But the acyl chains of the Pma1-interacting lipids (LP1–3) curved towards TMH3, TMH5, and TMH7 of Pma1. Their phosphate groups are stabilized by main chains of Y314 and I712, side chain of R315, and side chain of K781 of the neighbor Pma1 subunit (Fig. 2e). The resolution of the EM densities is insufficient to determine the chemical composition of the lipids, but the densities are incompatible with the presence of ergosterols or cholesterols. Previous work suggested that MCP is rich in sphingolipids that directly associate with Pma1^12,14–16^. Therefore, the observed lipids are likely a mixture of phospholipids and sphingolipids that are exported from the endoplasmic reticulum to the plasma membrane together with Pma1.

**Fig. 3:**
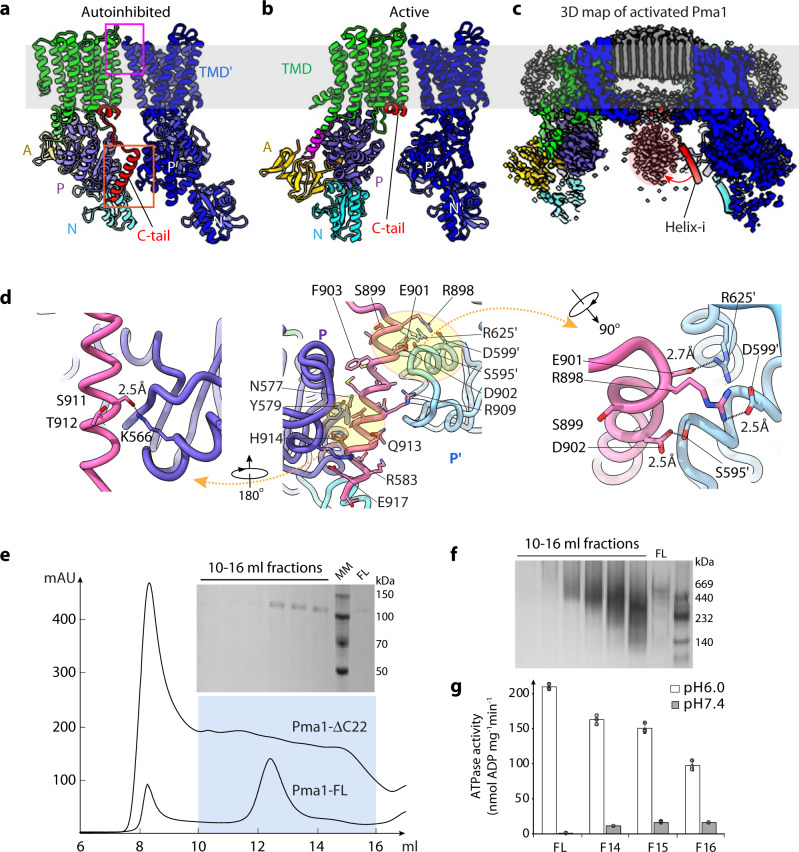
Mechanism of C-tail-mediated hexamer assembly and autoinhibition of Pma1. **a** Inter-subunit interactions of autoinhibited Pma1. Two neighboring Pma1 subunits are shown in multiple colors and blue, respectively. The interface on the exoplasmic side is highlighted by a magenta rectangle. The interface mediated by the C-tail is highlighted by an orange rectangle. **b** Inter-subunit interaction of activated Pma1. **c** Cut-in view of the activated Pma1 3D map displayed at a low threshold, showing a bulk of density corresponding to six oligomerized helices-i (pink oval). One autoinhibited Pma1 model was aligned to the map to show the movement of Helix-I. **d** Detailed interactions of the Helix-I with two neighboring P-domains of Pma1. **e** Gel filtration profiles of the full length (Pma1-FL) and Pma1-ΔC22. Inset is the SDS-PAGE gel of 10–16 ml fractions of Pma1-ΔC22. **f** The same fractions by native PAGE gel. Three independent experiments were conducted with similar results. **g** ATP hydrolysis activity at pH 6.0 and 7.4 of the full-length (FL) wild-type hexamer Pma1 and the truncated Pma1 in gel-filtration fractions F14 (elution volume 13–14 ml), F15 (14–15 ml), and F16 (15–16 ml). Each circle represents a data point. Data are represented as mean ± SD (*n* = 3).

The hexagonal lattice structure of the lipids is clearly imposed by the Pma1 hexamer. The protein-lipid interactions also enhance the subunit-subunit interface of the Pma1 hexamer. There are two major protein-protein interfaces: the above-mentioned interaction of the regulatory C-terminal helix with the P-domain of the neighboring protein in the cytosolic side, and the tight packing of TMH7 and TMH10 against the TMH3 and TMH4 of the neighboring Pma1 in the exoplasmic side to form a “V” shape (Fig. 3a, Supplementary Fig. 10). The second interface is extensive, involving polar residues T775, T776, K781, Q786, and R857 with Y314 and T316 of the neighboring subunit. This interface also contains extensive hydrophobic interactions. The exoplasmic TMD-TMD interface is stable in both autoinhibited and activated structures, accounting for the hexamer stability during the proton-pumping cycle. The unique architecture of “V” shape also indicates the lipids in cytosolic leaflet can easily diffuse away from the hexamer, which explains why only the lipids in exoplasmic leaflets are ordered in our structure.

### The Pma1 autoinhibition mechanism

As mentioned above, the inhibitory Pma1 C-tail contains the short horizontal helix and the longer helix-i which is sandwiched between two neighboring P-domains (Fig. 3a, d). Helix-i spans the last 22 residues (T897-T918) and contains three phosphorylation sites, S899, S911, and T912. In the autoinhibited state, S911, H914, and E917 of helix-i interact with K566, N577, Y579, and R583 of the P-domain to the left, and R898, E901, and D902 of helix-i interact with S595', D599', and R625' of the neighboring P-domain to the right. Major interactions include two inter-molecular salt bridges (R898:D599' and E901:R625') and an intramolecular hydrogen bond (S911:K566). At low pH, the two salt bridges will likely break up to liberate helix-i, leading to Pma1 activation. Phosphorylation of S899, S911, and T912 likely has the same effect as low pH in that it will break up the inhibitory interaction: phosphorylated S911 will prevent helix-i from binding to the P-domain, and phosphorylated S899 or T912 are right next to and will break up the two salt bridges.

As expected, the helix-i becomes disordered in the activated structure, enabling the movement of P-domains that are required during the ATPase cycle of Pma1. Interestingly, accompanying the disappearance of the helix-i is the emergence of a bulky density surrounded by the six cytosolic regions of the Pma1 hexamer, suggesting that the six helices-i liberated from the P-domains have moved to the center and interact with each other (Fig. 3b, c, Supplementary Fig. 9b). To examine this possibility, we expressed in and purified from *E. coli* the SUMO-tagged helix-i (C-terminal 22 residues). We found that the SUMO-tagged helix-i indeed oligomerized based on gel filtration elution volume and native gel electrophoresis (Supplementary Fig. 11). The C-tail helix-i oligomerization may prevent its rebinding to the P-domains and help to stabilize the activated Pma1 hexamer, in which the helix-i mediated inter-subunit interaction is absent.

We further examined the helix-i function both in vivo and in vitro. We found the helix-i truncated yeast strain (*Pma1-ΔC22*) was able to grow albeit much slower than wild-type yeast (Supplementary Fig. 12). We also produced the helix-i truncated Pma1 protein (Pma1-ΔC22) and found that the truncated Pma1 assembled heterogenous oligomers (Fig. 3e, f). The ATP hydrolysis activity of the Pma1-ΔC22 at pH 6.0 is reduced compared to the wild-type protein at pH 6.0, and the reduction is more significant for smaller oligomers (Fig. 3g). This observation indicates that the hexamer state of the wild-type protein is most efficient in ATP hydrolysis. However, unlike the wide-type Pma1 hexamer which has virtually no ATPase activity at pH 7.4, the truncated protein retained 10–15% ATPase activity at pH7.4 (Fig. 3g). The presence of significant residual ATPase at pH 7.4 of the truncated Pma1 is revealing and can be explained by two factors. First, the high pH (7.4) can no longer effectively inhibit the Pma1 ATPase activity in the absence of the helix-I, explaining the residual activity and highlighting the importance of the helix-i. Second, pH (proton) plays a dual function in Pma1: The pH (proton) not only regulates the helix-I binding but also functions as the substrate of the Pma1, which is a proton pump. In the absence of the helix-I in the truncated protein, the ATPase activity is expected to be stimulated by the substrate. This explains the 10–15% retained ATPase activity of the truncated protein at high pH (7.4).

### Conversion between autoinhibited and active forms may be cooperative

**Fig. 4:**
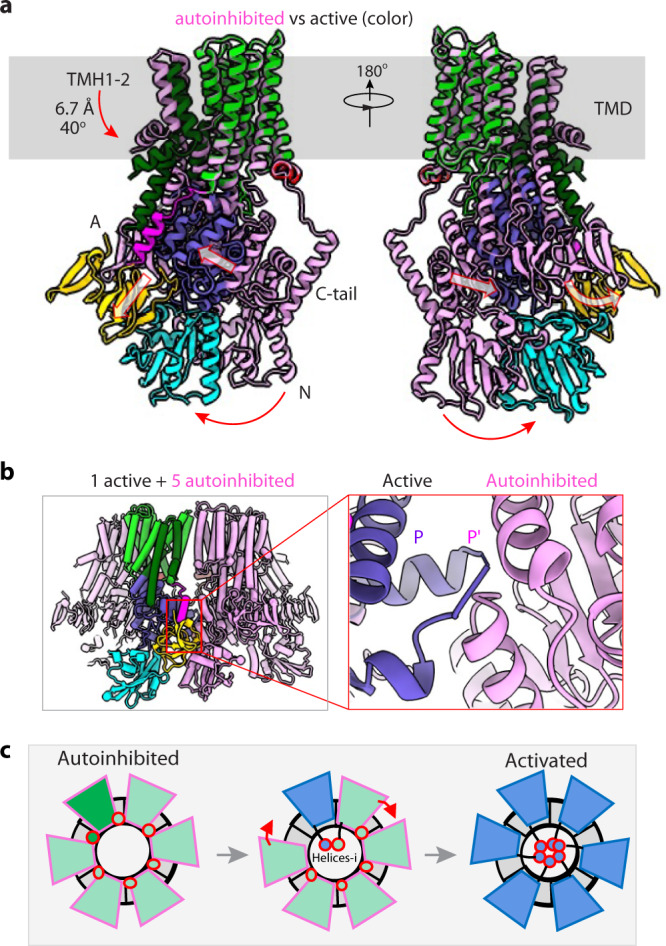
The cooperative model of the Pma1 hexamer. **a** Structural comparison of the Pma1 subunit in autoinhibited and activated E2P states. TMH1–2 move downward by 6.7 Å and rotate by 40°. **b** Structural model of the autoinhibited Pma1 hexamer, in which one protomer is replaced by the activated Pma1. The red rectangle highlights the region in which two neighboring P-domains would sterically clash. **c** A proposed cooperative activation model of the Pma1 hexamer. When one protomer in the Pma1 hexamer is activated, this protomer induces the activation of the next protomer by releasing its helix-i from the neighboring P-domain. Sequential release of the helices-i around the hexamer leads to the activation of all six protomers.

In the Post-Albers model that has been widely validated by structural and functional studies of numerous P-type ATPases, the ATPases cycle through four major states, E1-E1P-E2P-E2, in one cycle of ATP-hydrolysis-driven substrate transport^37^. We had obtained the Pma1 structure in the autoinhibited and active E2P states. We also generated a Pma1 homolog model in the E1-ATP state based on the C-terminal truncated monomeric AHA2 crystal structure in the ATP-bound E1 state^18^ (Fig. 4a, b, Supplementary Fig. 13). Comparison of the three Pma1 structures revealed dramatic conformational changes in the A, N, and P-domains. We found that TMH1, 2, and 4B moved, but TMH3, 4 A, and 5–10 were largely stationary among these structures. The positions of the P-domain in the autoinhibited and E1-ATP states were the same. Furthermore, the structural features of the modeled Pma1 in the E1-ATP state were consistent with its intermediate nature between the autoinhibited state and the active E2P state.

Compared to the autoinhibited state, the P-domain moves 30 Å toward TMH1–2, and the A- and N-domains move by 10 Å and 20 Å, respectively, toward each other in the E2P state. Because they are extensively connected to TMH1–2 and TMH4A, the movement of the A and P-domains pull TMH1–2 downward by 6.7 Å and turn these helices by 40°. These large conformational changes of TMH1–2 are likely related to Pma1’s proton pumping activity, as discussed below. Because TMH1–2 are at the outer periphery of the Pma1 hexamer, the proton-translocation movements per se of TMH1–2 (and TMH4B) in one Pma1 subunit appear to not affect those in the neighboring subunits.

However, structural alignment shows that a single active Pma1 subunit cannot be accommodated without severe steric conflict if the remaining five subunits are in the autoinhibited state (Fig. 4b). This indicates that activation of one subunit will sterically force its neighbor to take on the same active conformation (Fig. 4c). At the structural level, it seems likely that activation of the first subunit involves the release of the helix-i and a subsequent 30 Å move of the P-domain to the active position. Release of the helix-i will activate its left neighbor. The moved P-domain will push on the right neighboring P-domain, forcing it to move to the active position and dissociate from the inhibitory helix-i. Propagation of such conformational changes around the hexameric ring will lead to the activation of all six Pma1 subunits. Compared to the mechanism in which each protomer is activated independently, we surmise that the cooperative all-or-none activation mechanism is more rapid, which, in turn, may speed up yeast’s response to environmental stresses and ensure rapid fermentative growth^38,39^.

### A plausible proton translocation model

**Fig. 5:**
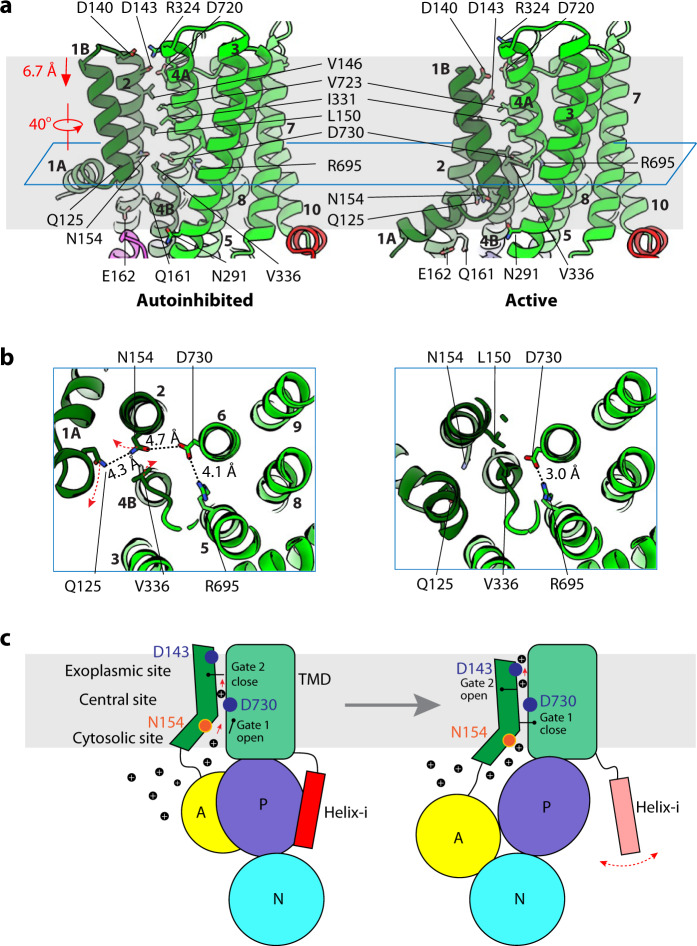
Substrate transport mechanism of Pma1. **a** Putative substrate transport path of Pma1 in the autoinhibited (left) and active state (right). TMH1–2 move downward by 6.7 Å and rotate by 40°. Residues lining the path are shown as sticks. **b** Cut-in view from the exoplasmic side of the central proton binding site around D730 in the autoinhibited (left) and active state (right). The section plane is the cyan plane in (**a**). **c** A model for proton transport across the plasma membrane by Pma1. More details are in the text.

During the transition between Pma1’s autoinhibited and activated states, the movement of TMH1–2 with respect to the remaining TMD region suggests that protons are likely transported between them (Fig. 5a, b, Supplementary Fig. 9c–f). This substrate transport path is shared among many P-type ATPases characterized so far^4,40–42^. The Pma1 transport path features three groups of polar/charged residues: (1) R695 and D730 in the middle of the membrane bilayer; (2) D140, D143, R324, and D720 in the exoplasmic half of the membrane; and (3) Q125, N154, Q161, and E162 in the cytosolic half of the membrane. The three hydrophilic/charged groups are separated by two groups of hydrophobic residues: the upper group near the exoplasmic side contains V146, L150, I331, and V723, and the lower group near the cytosolic side contains V336 (Fig. 5a, b). Most of these Pma1 residues, including D143, V146, L150, N154, Q161, E162, V336, and D730, are conserved in the plant AHA2 (Supplementary Fig. 14). The three hydrophilic/charged groups are likely involved in proton transport. D730, which is in the middle of the membrane, is the only acidic residue in the proton transport path. The D730 equivalent in the plant AHA2 (D684) was shown to be essential for proton transport^43,44^. Pma1 proteins containing D730 or R695 mutations were shown to lose ATPase activity^45^. In addition, previous studies show that the D143 mutants were lethal^46,47^. The corresponding residue D138 in *S. pombe* Pma1 was shown to be important for Pma1 activity and glucose uptake^48^.

In the autoinhibited state, D730 is surrounded by R695, N154, and Q125 but these residues are outside the range of direct interaction. These residues may have lowered the pKa of D730 sufficiently that D730 is likely protonated. This middle hydrophilic/charged group is connected to the cytosolic proton entry point (Fig. 5a, b). However, the middle group is separated by the upper hydrophobic group from the potential proton-relaying residues D140, D143, and D720 in the exoplasmic side. Indeed, D143 forms a salt bridge with R324 and is not able to transfer protons. Therefore, in the autoinhibited state, D730 is likely protonated, and the proton transfer pass is blocked between D730 and the exoplasmic side but is continuous between D730 and cytoplasm.

In the active state, N154 and Q125 move downward by 6.5 Å from their positions in the autoinhibited state to face the cytosolic solvent and attract protons. D730 forms a salt bridge with R695. Therefore, D730 must be deprotonated now (Fig. 5a, b). The upper hydrophobic group, in particular L150, now shifts away and no longer blocks the proton path from D730 to D143 in the exoplasmic side. Indeed, D143 moves by ~7.5 Å toward D730 to accept protons from D730. The lower hydrophobic group—V336 in the kink region of TMH4A and TMH4B—now blocks the proton translocation path between D730 and N154 in the cytosolic side. The V336 blockade likely prevents the protons released by D730 from backtracking to the cytosolic side. Therefore, in the active E2P state, D730 is likely deprotonated, and the proton transfer path is open between D730 and the exoplasmic side but is blocked between D730 and the cytoplasm.

Based on these results, we propose the conformational change-associated proton transport model of Pma1 (Fig. 5c, Supplementary Movie 1). The conformational changes in TMH1–2 are driven by the 30 Å movement of the A-domain, which is triggered by ATP hydrolysis in the Pma1 cytosolic domains. We suggest that the largely hydrophilic interface between TMH1–2 and TMH4–6 is where the proton translocation occurs. In this model, D730 at the middle of the lipid bilayer takes the center stage and is protonated and deprotonated in each transport cycle. There are two hydrophobic gates: the upper gate is located between D730 and the exoplasm, and the lower gate is between D730 and the cytosol. These two gates open and close alternatively to ensure the outward directionality of the proton movement. Protonation of D730 is facilitated by the close approaches of N154 and Q125 that lower the pKa, and D730 deprotonation is induced by the movement of N154 and Q125 away from D730 by 6.5 Å and the subsequent salt bridge formation between D730 and R695. In this scenario, the group of hydrophilic/charged resides at the cytosolic side helps to attract protons from the cytoplasm, and the group of hydrophilic/charged residues at the exoplasmic side helps to expel the protons into the extracellular milieu.

A previous study summarized 11 important point mutations in the transmembrane region of Pma1 that either was lethal or drastically diminished the ATPase activity^45^. By mapping these residues on our structures, we found that they are largely clustered around the exoplasmic half of the proton transport path (Supplementary Fig. 15), suggesting a tight coupling between the proton translocation and the ATP hydrolysis activity.

## Discussion

In yeast cells, the MCP is a network-like microdomain structure that surrounds punctate MCC microdomains in the plasma membrane^49^. Exocytosis and endocytosis in yeast exclusively originate from the MCP structure^50,51^. These membrane domains have been studied by fluorescent microscopy, revealing their approximate size and morphology^9–11,52,53^. But the ultrastructural details have been lacking. Based on our surprising observation that the Pma1 hexamer encloses ~57 ordered lipids, we suggest that this protein-lipid complex structure is the major building block of the *S. cerevisiae* MCP. The ordered and protein-demarcated nature of these lipids may be one mechanism that accounts for their impeded lateral diffusion in these microdomains^54^. How the building blocks associate to form an MCP network is currently unknown. Super-resolution microscopy has revealed that the MCP is likely made of isolated foci^52^. It is possible that these foci correspond to individual Pma1 hexamers or to small clusters of Pma1 hexamers. But lipids or other proteins such as Hxt1, a glucose transporter, are also likely to contribute to the MCP network assembly^53^. The 57 ordered lipids we observed are in the outer leaflet of the membrane. But we also observed a cloudy density in the cytoplasmic leaflet side of the interior of the Pma1 hexamer; this weaker density is likely from disordered lipid molecules. The disorder of the inner leaflet may be due to the changing available space as Pma1 goes through the proton pumping cycle.

The hexagonal lipid lattice was not observed in the *Neurospora carassa* Pma1 structure^35^. It is unclear if this is due to a difference in the sample preparation procedure or due to a difference in the MCP structure in different fungal species. In this regard, the structure and compositions of the *Neurospora* MCC/eisosomes were found to be quite different from their *S. cerevisiae* counterparts^55^. In fact, there have been no reports describing the existence of the MCP structure in *Neurospora*.

The *Neurospora* Pma1 structure was captured only in the inhibited state^35^. We have captured the *S. cerevisiae* Pma1 structure in both inhibited and active states. Our structures have revealed large conformational changes induced by ATPase activation and ATP hydrolysis, elucidated molecular mechanisms for the protein’s autoinhibition and activation, and suggested a plausible mechanism for proton export by Pma1. We have shown that autoinhibition of Pma1 is achieved primarily by the C-tail helix-i simultaneously binding and constraining the movement of two neighboring P-domains. Despite a lack of phosphorylation in our preparation, our use of low pH (pH 6) and BeF_3_^−^ successfully converted the autoinhibited Pma1 to the active E2P state in which all six C-tail helices-i of the Pma1 hexamer are liberated from the P-domains and oligomerize to sustain the activated state and stabilize the hexamer architecture. Compared to Pma1, the plant AHA2 has a longer C-tail, which interacts with the 14-3-3 complex. Therefore, the activation and regulatory mechanism of AHA2 are probably different and await further study.

Because of the large structural difference between the inhibited and activated states, the hexamer architecture is incompatible with the coexistence of inhibited and activated subunits in the same hexamer ring. This leads us to suggest that Pma1 activation is a cooperative all-or-none process in which all subunits are in an active or inactive state. This model also enables the proposal of a more detailed proton translocation mechanism. A similar proton pumping mechanism was previously proposed based on the crystal structure of the inhibitory C-terminal helix truncated and monomeric Arabidopsis AHA2 in a single nucleotide-binding state, and key residues such as the AHA2 D684 (D730 in Pma1) and N106 (N154 in Pma1) were already identified^18,34^. However, our mechanism is based on the experimental structures of the full-length Pma1 in the functional hexameric form and in two conformations. Therefore, the current mechanism is more detailed with several novel features: (1) Only TMH1/2/4B move while other TMHs are stationary in our proton pumping model; (2) In addition to the previously reported D730 and N106, R695 in the center and D143 and R324 in the extracellular side are identified as playing important roles in proton transfer; (3) Two hydrophobic gates are identified along the proton path; (4) Our mechanism couples the movement of the cytosolic A/N/P-domains with the proton transfer from the cytosol to extracellular side.

Capturing Pma1 in more states during the Post-Albers cycle will undoubtedly provide additional insights into the ATP-hydrolysis-driven proton pumping mechanism. Finally, given that Pma1 is essential in fungi but absent in mammalian cells, Pma1 is an ideal target for the development of antifungal therapeutics^56–59^. The structures described here will facilitate these endeavors.

## Methods

### Expression and purification of Pma1

We tagged Pma1 with a C-terminal triple-FLAG in the yeast strain BY4742 using a PCR-based genomic epitope-tagging method^60^. The primers are listed in Supplementary Table 2. Cells were first grown in 200 mL SD-H medium for about 20 h, then transferred to 9 L of YPD medium for another 24 h before harvest. Cells were resuspended in lysis buffer (20 mM Tris-HCl, pH 7.4, 0.2 M sorbitol, 50 mM potassium acetate, 2 mM EDTA, and 1 mM phenylmethylsulfonyl fluoride [PMSF]) and then lysed using a French press at 15,000 psi. We centrifuged the lysate at 10,000 × *g* for 30 min at 4 °C, and collected the supernatant for another centrifuge cycle at 100,000 × *g* for 60 min at 4 °C. The membrane pellet was collected and then resuspended in buffer A containing 10% glycerol, 20 mM Tris-HCl (pH 7.4), 1% DDM, 0.1% cholesteryl hydrogen succinate [CHS], 500 mM NaCl, 1 mM MgCl_2_, 1 mM EDTA, and 1 mM PMSF. After incubation for 30 min at 4 °C, the mixture was centrifuged for 30 min at 100,000 × *g* to remove the insoluble membrane. We loaded the supernatant into a pre-equilibrated anti-FLAG (M2) affinity column (GenScript) at 4 °C and washed the affinity gel with buffer B (20 mM HEPES, pH 7.4, 150 mM NaCl, 0.01% lauryl maltose neopentyl glycol [LMNG], 0.001% CHS, and 1 mM MgCl_2_). The proteins were eluted with buffer B containing 0.15 mg/mL 3 × FLAG peptide and were further purified in a Superose 6 10/300 Increase gel filtration column in buffer C (20 mM HEPES, pH 7.4, 150 mM NaCl, 0.003% LMNG, 0.0003% CHS, and 1 mM MgCl_2_). For samples in pH 6.0, the gel filtration buffer C was replaced by buffer D (20 mM MES, pH 6.0, 150 mM NaCl, 0.003% LMNG, 0.0003% CHS, and 1 mM MgCl_2_). Finally, the purified proteins were assessed by SDS-PAGE gel and concentrated for cryo-EM analysis.

### Cryo-electron microscopy

To capture different states, the purified Pma1 in pH 7.4 or pH 6.0 were used for EM grid preparation directly or mixed with E2P solution (5 mM MgCl_2_, 10 mM NaF, and 2 mM BeSO_4_) for 1 h on ice. After incubation, 2.5 μL aliquots of Pma1 at a concentration of about 3 mg/mL were placed on glow-discharged holey carbon grids (Quantifoil Au R2/1, 300 mesh) and were flash-frozen in liquid ethane using a FEI Vitrobot Mark IV. Cryo-EM data was collected automatically with SerialEM in a 300-kV FEI Titan Krios electron microscope with defocus values ranging from −1.0 to −2.0 μm. The microscope was operated with a K3 direct detector at a nominal magnification of ×130,000. The total doses were 50–60 electrons per Å^2^ at the sample level.

### Cryo-EM image processing

We used the program MotionCorr-2.0^61^ for motion correction, and CTFFIND-4.1^62^ for the calculation of contrast transfer function parameters. We used RELION-3^63^ for all remaining steps. The resolution of the map was estimated by the gold-standard Fourier shell correlation at a correlation cutoff value of 0.143.

For the wild-type autoinhibited Pma1 structure in pH 7 (Pma1-pH7), we collected 4152 raw movie micrographs. A total of 676,610 particles were picked automatically for 2D and 3D classifications. Based on the quality of the four 3D classes, 179,392 particles were retained for further refinement and postprocessing, resulting in a 3.2-Å average resolution 3D map using C1 symmetry and a 2.9-Å average resolution 3D map using C6 symmetry.

For the Pma1 structure in pH 7.0 and incubated with BeF_3_^−^ (Pma1-pH7-BeF), we collected 2910 raw movie micrographs. A total of 413,357 particles were picked automatically for 2D and 3D classifications. Based on the quality of the four 3D classes, 107,231 particles were retained for further refinement and postprocessing, resulting in a 3.8-Å average resolution 3D map using C1 symmetry.

For the wild-type Pma1 structure in pH 6 (Pma1-pH6), we collected 3436 raw movie micrographs. A total of 493,148 particles were picked automatically for 2D and 3D classifications. Based on the quality of the four 3D classes, 110,159 particles were retained for further refinement and postprocessing, resulting in a 3.8-Å average resolution 3D map using C1 symmetry.

For the Pma1 structure in pH 6 and incubated with BeF_3_^−^ (Pma1-pH6-BeF), we collected 3492 raw movie micrographs. A total of 518,281 particles were picked automatically for 2D and 3D classifications. Based on the quality of the four 3D classes, one class in activated conformation with 63,661 particles (Pma1-pH6-BeF-Conf1) and another class in autoinhibited conformation with 122,922 particles (Pma1-pH6-BeF-Conf2) were retained for further refinement and postprocessing, respectively. Finally, the activated Pma1-pH6-BeF-Conf1 map was refined to 3.8-Å average resolution using C1 symmetry and 3.4-Å average resolution using C6 symmetry; the autoinhibited Pma1-pH6-BeF-Conf2 map was refined to 3.8-Å average resolution using C1 symmetry.

For the Pma1 structure in 500 mM NaCl and pH 6.0 (Pma1-pH6-high salt), we collected 242 raw movie micrographs. A total of 82,446 particles were picked automatically for 2D and 3D classifications. Based on the quality of the three 3D classes, 15,816 particles were retained for further refinement and postprocessing, resulting in a 3.6-Å average resolution 3D map using C6 symmetry.

### Structural modeling, refinement, and validation

We first built the model of Pma1 in the autoinhibited state at 3.2 Å and 2.9 Å resolution (Pma1-pH7-C1, Pma1-pH7-C6). We generated the initial model based on the structure of plant AHA2 (PDB ID 5KSD) by SWISS-MODEL (https://swissmodel.expasy.org), and then manually corrected it in COOT^64^ and Chimera^65^. Because of the limited local resolution, the A- and N-domains of Pma1 were only fitted into the density by rigid body fitting. The complete Pma1 model was refined by real-space refinement in the PHENIX program^66^ and subsequently adjusted manually in COOT. Using the autoinhibited model of Pma1 as a reference, the model of activated Pma1 was built into the active E2P map of Pma1 (Pma1-pH6-BeF-conf1) and refined using COOT, Chimera, and PHENIX. Finally, all models were validated using MolProbity^67^. Structural figures were prepared in Chimera and PyMOL (https://pymol.org/2/).

### Expression and purification of C-terminal helix-I peptide

The C-terminal 22 amino acids of Pma1 were fused with a 6×His-SUMO tag and TEV cleavage protease site at the N-terminus by cloning into the pET28a vector. The construct was then transformed into Rosetta (DE3), and the protein was expressed by induction of IPTG. The protein was purified by Ni-beads in buffer A (20 mM HEPES, pH 7.4, 500 mM NaCl, 10% glycerol), and Superdex 200 10/300 gel filtration in buffer B (20 mM HEPES, pH 7.4, 150 mM NaCl). The 6×His-SUMO control sample was obtained by removing the peptide using TEV protease treatment.

### Expression and purification of Pma1-△C22

Similar to our procedure with wild-type Pma1, the Pma1-△C22 was first cloned in the yeast strain BY4742 using a PCR-based genomic epitope-tagging method. However, this strain only yielded a small amount of purified protein. Therefore, we cloned the Pma1-△C22 into the PRS423 vector with a FLAG tag at its C- terminus and transformed the construct into the BY4742 yeast strain. Purification of Pma1-△C22 followed the same protocol of the wide type Pma1 mentioned above.

### ATP hydrolysis assay

The ATPase activity assays were performed using the ADP-Glo kit (Promega) to measure free ADP in solution. Each reaction contained 0.16 μg purified protein, 100 mM HEPES 7.4 or 100 mM MES 6.0, 150 mM NaCl, 10 mM MgCl_2_, 0.003% LMNG, 0.0003% CHS, and 0.5 mM ATP in a total volume of 5 μL. The mixture was first incubated for 30 min at 37 °C, and 5 μL of ADP-Glo reagent was then added. Then the mixture was incubated for 40 min at room temperature, and 10 μL of ADP-Glo detection reagent added. After 60 min, luminescence was detected by Synergy H1 Hybrid Multi-Mode Microplate Reader (BioTek).

### Native PAGE

Native PAGE was carried out on an electrophoresis system with power supply (BEIJING LIUYI BIOTECHNOLOGY CO., LTD.) under nondenaturing conditions in gels (1.5 mm thickness) containing 4–20% polyacrylamide. Native PAGE was carried out in electrophoresis buffer (25 mM Tris and 192 mM glycine, pH 8.8) at 100 V for 1.5 h.

### Colony growth assay

The wild-type yeast strain and the Pma1-△C22 strain were first grown in YPD medium at 30 °C overnight to the same OD. Then 7 μL of 1:10 serial dilutions of the cells were spotted onto YPD plates, incubated at 30 °C for 2 days, and examined for growth.

### Reporting summary

Further information on research design is available in the Nature Research Reporting Summary linked to this article.

## Supplementary information

Supplementary Information

Description of Additional Supplementary Files

Supplementary Movie 1

Reporting summary

## Data Availability

The cryo-EM 3D maps and the corresponding atomic models of the Pma1 have been deposited at the EMDB database and the RCSB PDB with the respective accession codes of EMD-31986 and 7VH5 (Pma1-pH7-C1), EMD-31987 (Pma1-pH7-C6), EMD-31988 and 7VH6 (Pma1-pH6-BeF-Conf1-C1), EMD-31989 (Pma1-pH6-BeF-Conf1-C6), EMD-31990 (Pma1-pH6-BeF-Conf2), EMD-31991 (Pma1-pH6-C1), EMD-31992 (Pma1-pH6-high salt), and EMD-31993 (Pma1-pH7-BeF). Source data are provided with this paper.

## Source data

Source Data
