# Supplementary material for: Structure and activation mechanism of the hexameric plasma membrane H+-ATPase
Source: Nat Commun. 2021 Nov 8;12:6439. doi: 10.1038/s41467-021-26782-y (PMC8575881; doi:10.1038/s41467-021-26782-y)
Supplement: Supplementary file 1 — Supplementary Information [file 41467_2021_26782_MOESM1_ESM.pdf]

Supplementary information for

Structure and activation mechanism of the hexameric plasma membrane H^+-ATPase

By Zhao et al.

This document contains 2 supplementary tables and 15 supplementary figures.

Supplementary Table 1. Cryo-EM data collection, 3D reconstruction and modeling

Sample	***S. cerevisiae*** **Pma1**							
Datasets	**pH7.4**		**pH7.4 + BeF_3^-**	**pH6.0**	**pH6 + BeF_3^-**			**pH6.0+500mM NaCl**
Map and Model	**Pma1-pH7-C1**	**Pma1-pH7-C6**	**Pma1-pH7-BeF**	**Pma1-pH6**	**Pma1-pH6-BeF-Conf1-C1**	**Pma1-pH6-BeF-Conf1-C6**	**Pma1-pH6-BeF-Conf2**	**Pma1-pH6-highsalt**
**Data collection and processing**								
Microscope	FEI Titan Krios							
Voltage (kV)	300							
Electron exposure ($e^-/Å^2$)	65		50	50	50			50
Defocus range (-μm)	1.0-2.0		1.0-2.0	1.0-2.0	1.0-2.0			1.0-2.0
Pixel size (Å)	0.826		1.04	1.04	1.04			1.04
Micrographs (no.)	4152		2910	3436	3492			242
Initial particle images (no.)	676,610		413,357	493,148	518,281			82,446
Final particle images (no.)	179,392		107,231	110,359	63,661		122,922	15,816
Symmetry imposed	C1	C6	C1	C1	C1	C6	C1	C6
Map resolution (Å)<br>FSC threshold	3.2<br>0.143	2.9<br>0.143	3.8<br>0.143	3.8<br>0.143	3.8<br>0.143	3.4<br>0.143	3.8<br>0.143	3.6<br>0.143
Map resolution range (Å)	250 - 3.2	250 – 2.9	250 - 3.8	250 - 3.8	250 - 3.8	250 - 3.4	250 - 3.8	250 - 3.6

**Refinement**								
Map sharpening *B* factor (Å^2)	245.6	100.8	140.6	161.7	129.0	132.4	159.2	92.4
Model composition<br>Non-hydrogen atoms<br>Protein residues<br>Lipids	39976<br>4830<br>58				38292<br>4620<br>58			
R.m.s. deviations<br>Bond lengths (Å)<br>Bond angles (°)	0.01<br>0.96				0.01<br>1.16			
Validation<br>MolProbity score<br>Clash score<br>Poor rotamers (%)	2.02<br>14.2<br>0				2.16<br>13.6<br>0			
Ramachandran plot<br>Favored (%)<br>Allowed (%)<br>Disallowed (%)	95.85<br>4.15<br>0				96.09<br>3.91<br>0			

Supplementary Table 2. Primers used in the study

Primer name	Sequence
Pma1up	ACTTCATGGCTGCTATGCAAAGAGTCTCTACTCAACACGAAAAGGAAACCCGGATCCCCGGGTTAATTAA
Pma1down	AGTTGATTAAAATGTGACAAAAATTATGATTAAATGCTACTTCAACAGGATGAATTCGAGCTCGTTTAAAC
Pma1up-CT	AAGCCTTTGACAGATTGATGAACGGTAAGCCAATGAAGGAAAAGAAGTCTCGGATCCCCGGGTTAATTAA

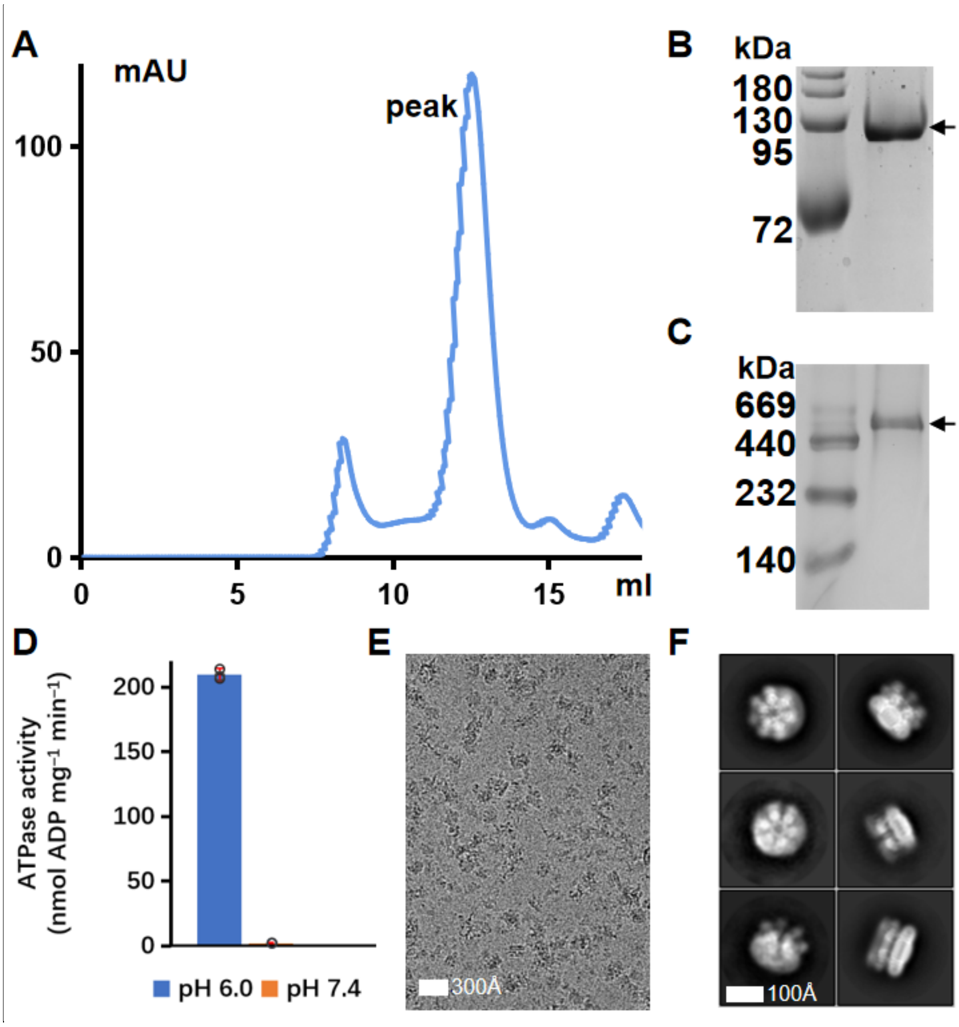

Supplementary Figure 1. Purification and analysis of Pma1. A) Gel filtration profile of Pma1. The peak fraction was checked by Coomassie blue-stained SDS-PAGE gel (**B**) and native PAGE gel (**C**). Three independent experiments were conducted with similar results. **D**) ATP hydrolysis activity of Pma1 in pH 6.0 and 7.4. **D**) ATP hydrolysis activity of Pma1 in pH 6.0 and 7.4. Each circle represents a data point. Each circle represents a data point. Data are represented as mean ± SD (n=3). **E**) A representative electron micrograph. **F**) Selected reference-free 2D class averages.

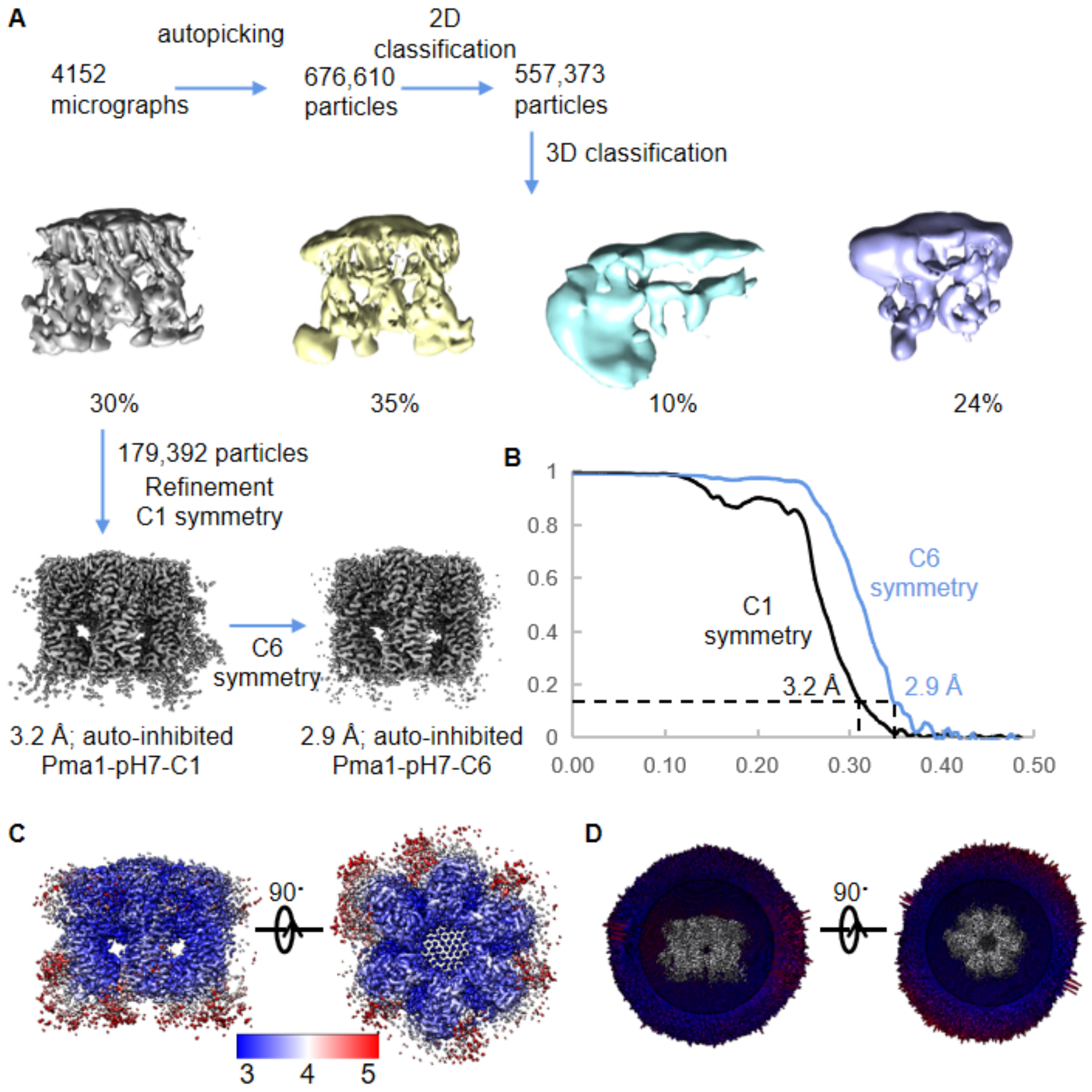

Supplementary Figure 2. Cryo-EM data processing and resolution estimation of Pma1 in the autoinhibited state. A) Cryo-EM data processing procedure. **B**) Gold-standard Fourier shell correlation of two independent half 3D maps of autoinhibited Pma1 in C1 and C6 symmetry. **C**) Local resolution map of the 3.2-Å 3D map of Pma1 in C1 symmetry. **D**) Angular distribution of raw particles used in 3D reconstruction of the 3.2-Å 3D map of Pma1 in C1 symmetry.

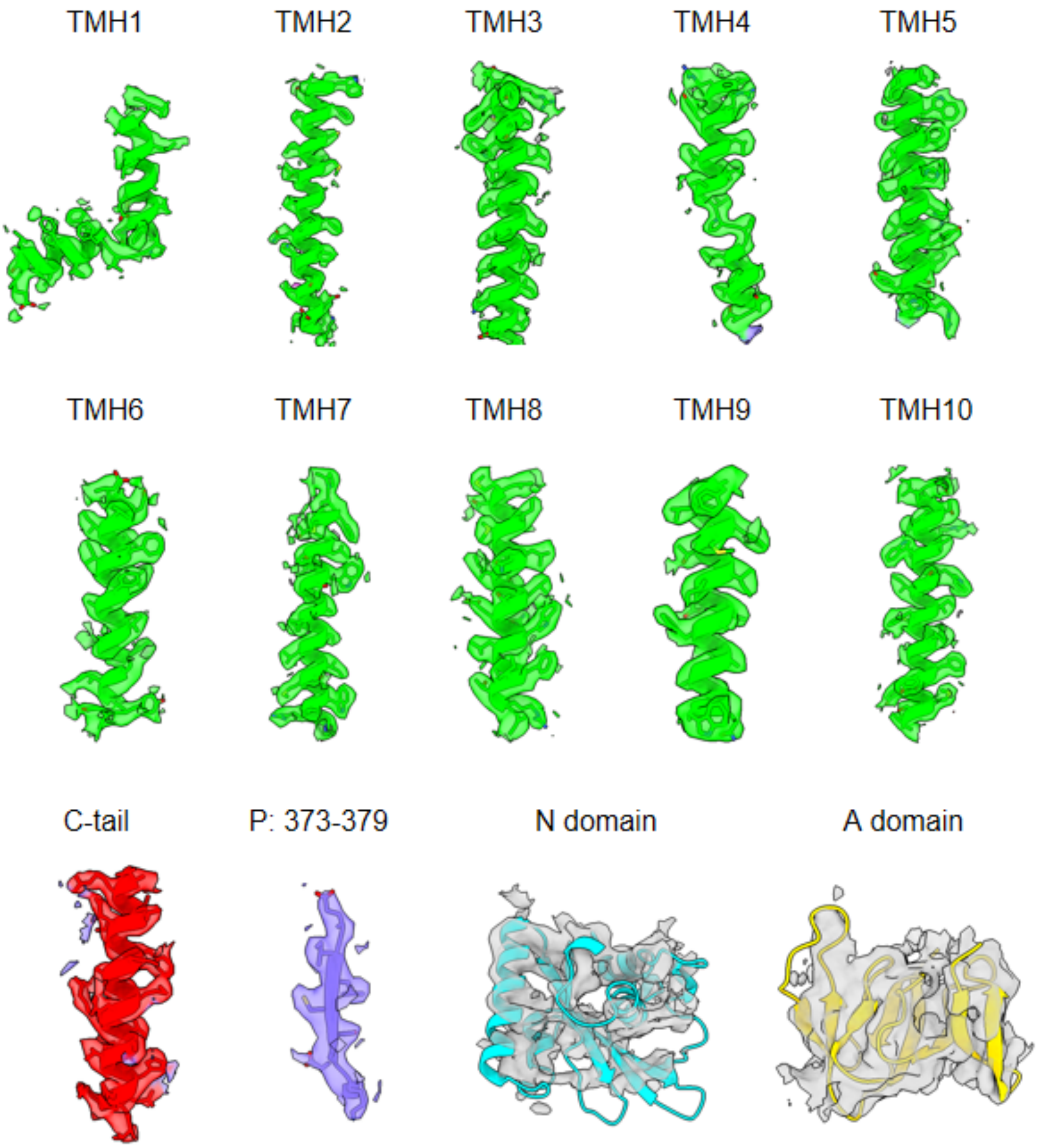

Supplementary Figure 3. Selected regions in the 3D map of Pma1-pH7-C1 in the auto-inhibited state at the resolution of 3.2 Å, superimposed on the atomic model.

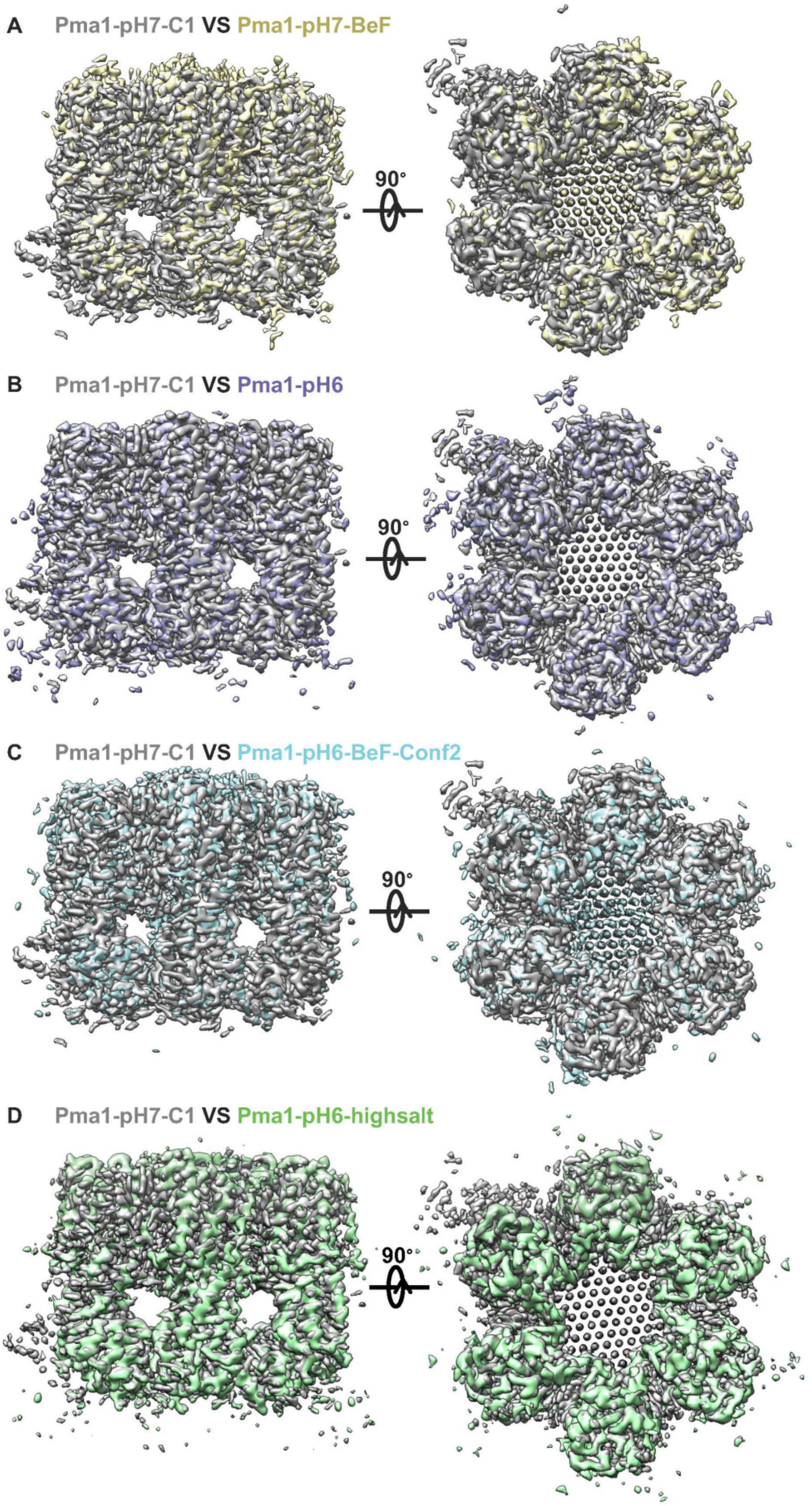

Supplementary Figure 4. Superimposition of the Pma1-pH7-C1 map with that of the Pma1-pH7-BeF (A), with the Pma1-pH6 (B), with the Pma1-pH6-BeF-Conf2 (C), and with the Pma1-pH6-highsalt (D). These four 3D maps are very similar.

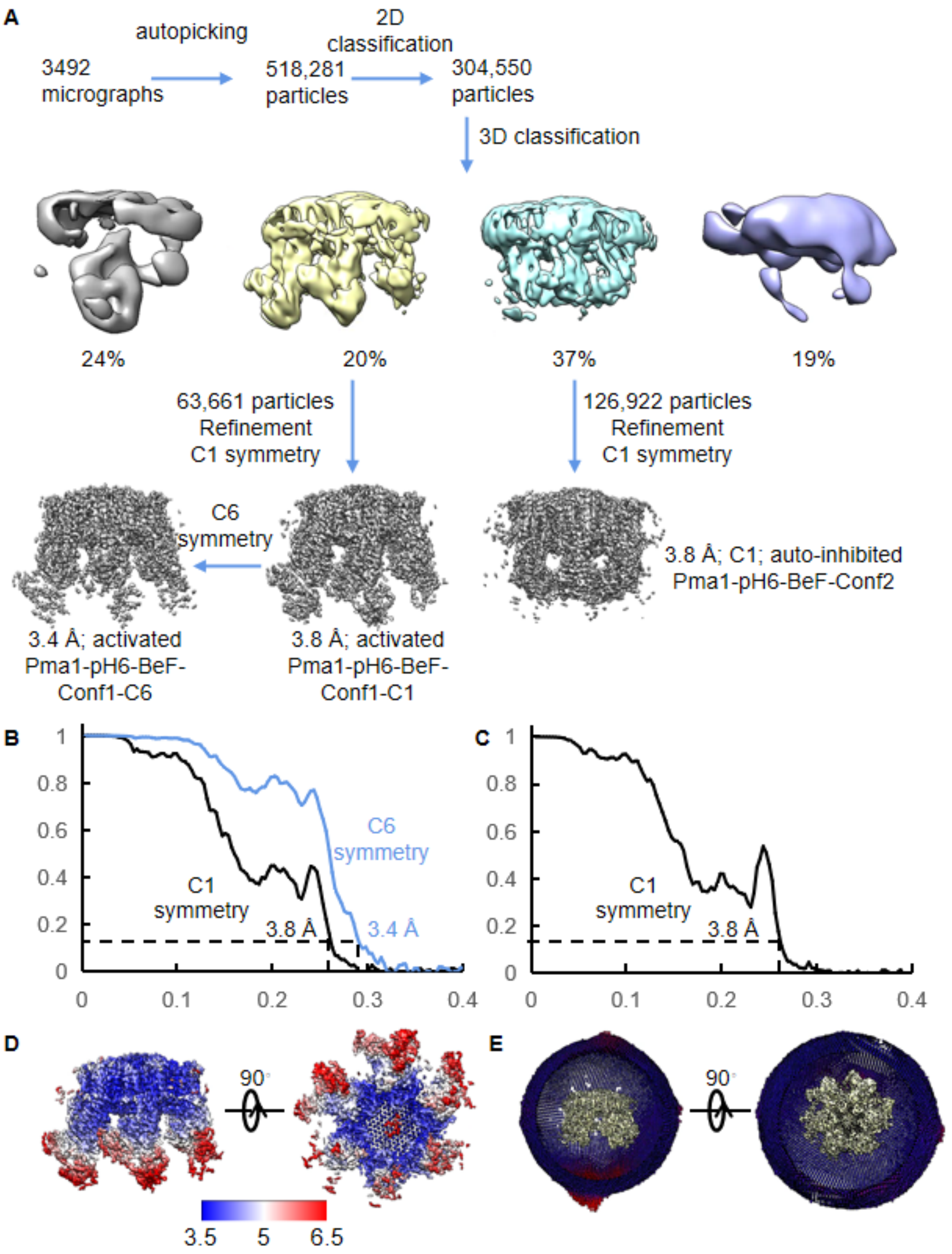

Supplementary Figure 5. Cryo-EM data processing and resolution estimation of Pma1 in the activated state. A) Cryo-EM data processing procedure. **B**) Gold-standard Fourier shell correlation of two independent half 3D maps of activated Pma1 in C1 and C6 symmetry. **C**) Gold-standard Fourier shell correlation of two independent half 3D maps of autoinhibited Pma1 in C1 symmetry. **D**) Local resolution map of the 3.8-Å 3D map of activated Pma1 in C1 symmetry. **E**) Angular distribution of raw particles used in 3D reconstruction of the 3.8-Å 3D map of activated Pma1 in the C1 symmetry.

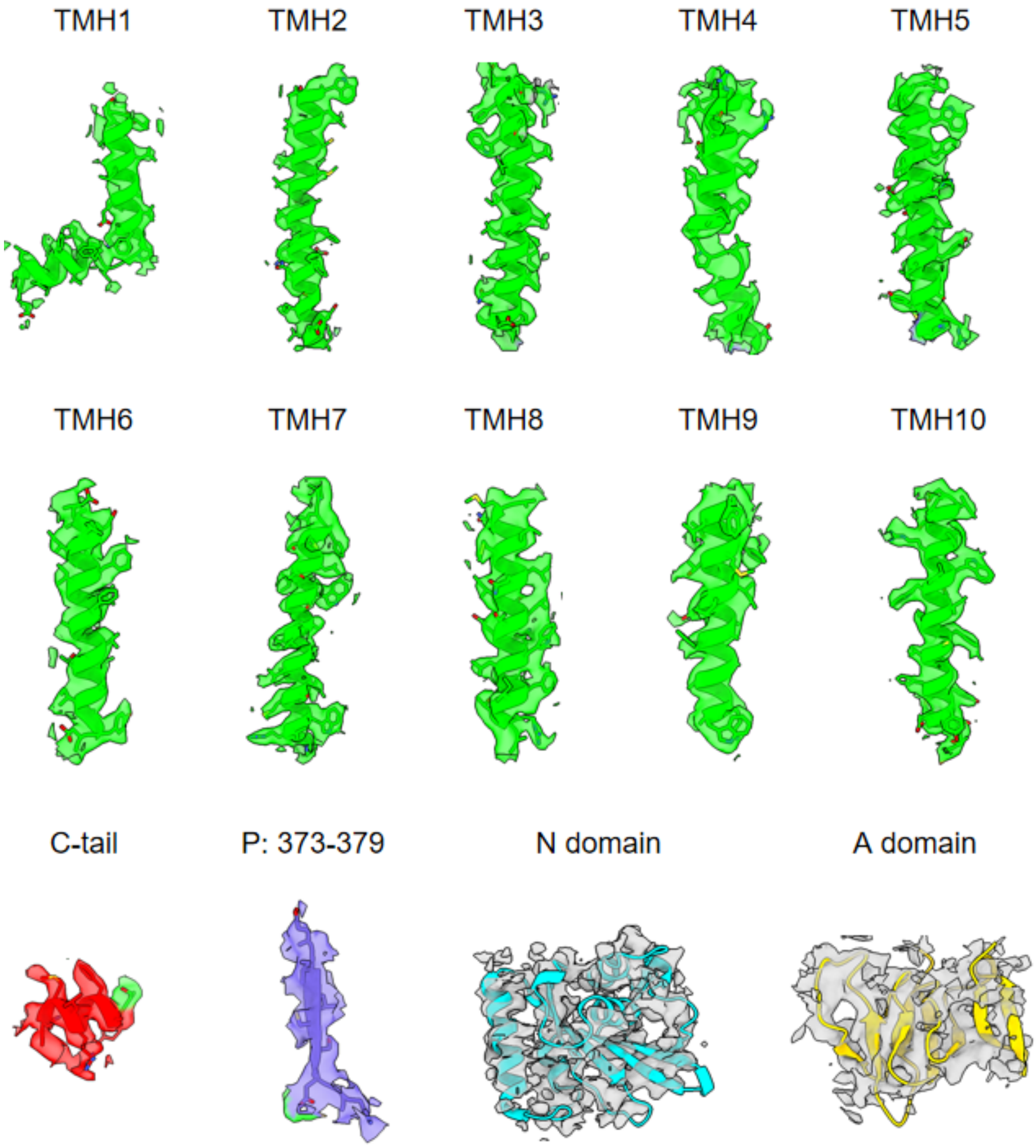

Supplementary Figure 6. Selected regions in the 3D map of Pma1-pH6-BeF-Conf1 in the activated state at the resolution of 3.8 Å, superimposed on the atomic model.

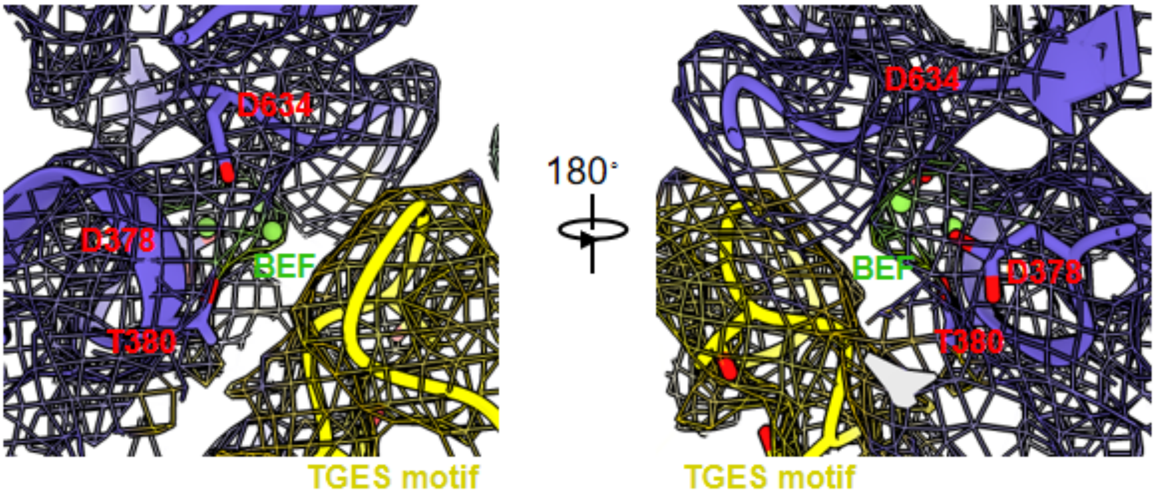

Supplementary Figure 7. The EM density around the BeF_3^-. The density is of the activated Pma1-pH6-BeF-Conf1 state and is shown as wires. BeF_3^- is shown as green spheres and labeled BEF.

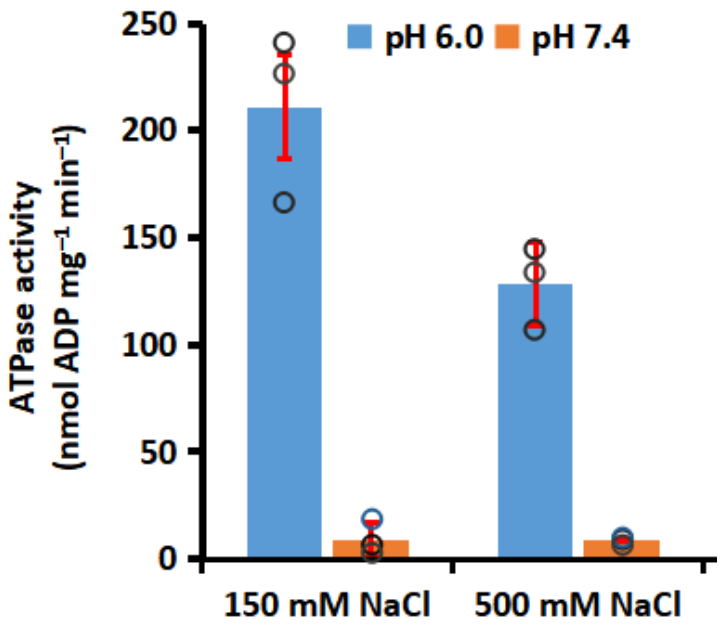

Supplementary Figure 8. ATP hydrolysis activity of Pma1 in buffer containing 150mM or 500mM NaCl at pH 6.0 or 7.4. Each circle represents a data point. Data are represented as mean ± SD (n=3).

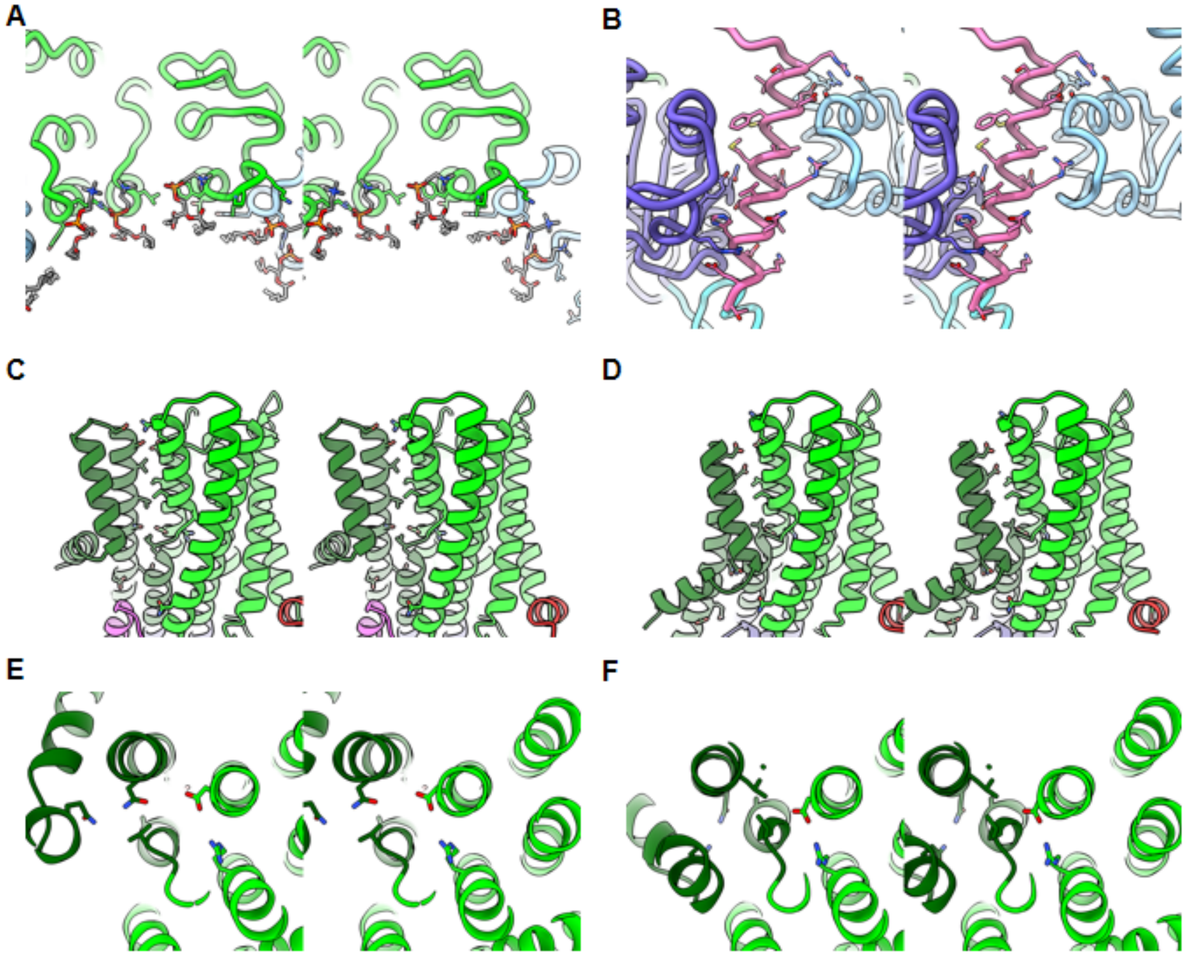

Supplementary Figure 9. Stereo views of selected regions highlighted in the main figures. A) The interface between Pma1 and bound lipid molecules. This interface is also shown in Fig. 2e. **B)** The C-terminal inhibitory helix. Also shown in Fig. 3d. **C and E)** show the stereo views of the substrate transporting path in the autoinhibited structure in a side and a top view. This region is also shown in Fig. 5a-b. **D and F)** Stereo views of the substrate transporting path in the active E2P structure in a side and a top view. This region is also shown in Fig. 5a-b.

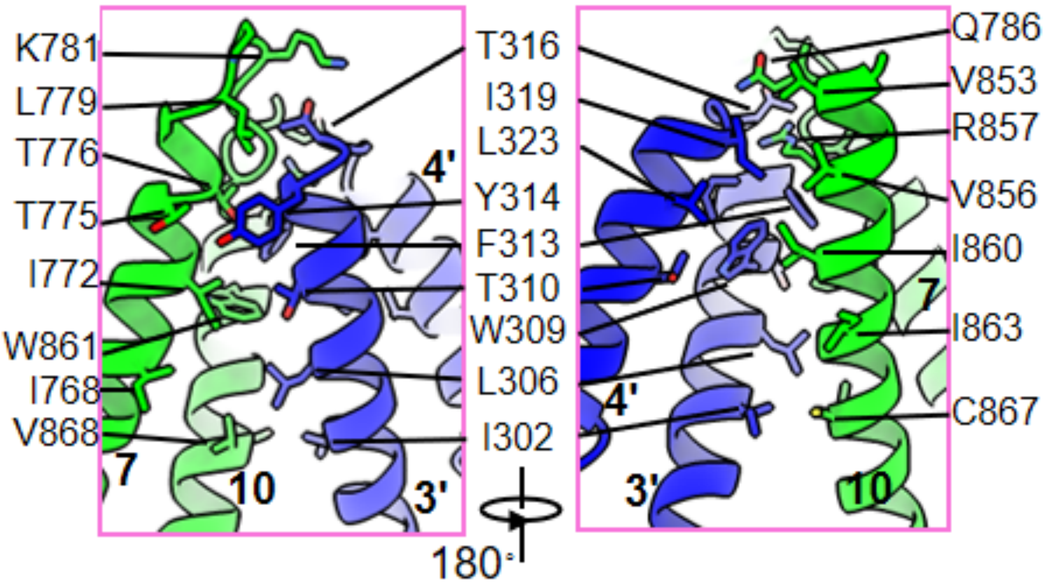

Supplementary Figure 10. Subunit interface in the exoplasmic side of Pma1 hexamer.

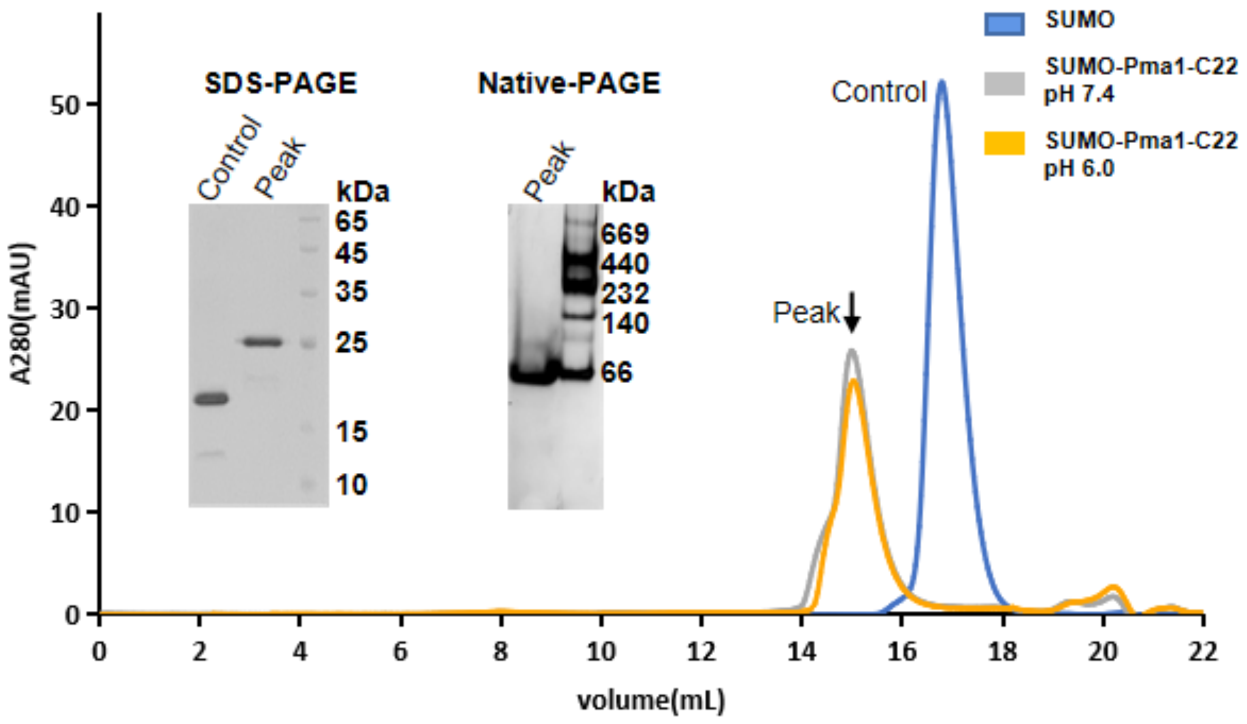

Supplementary Figure 11. The truncated Pma1 C-terminal Helix-I (C22) oligomerizes in solution. Superposition of the gel filtration profiles of the purified Sumo-tagged Pma1 Helix-I truncation at pH 7.4 (gray) and 6.0 (orange). Inserted are the SDS-PAGE gel image (left) and the native PAGE gel image (right) of the gel filtration peaks. Three independent experiments were conducted with similar results.

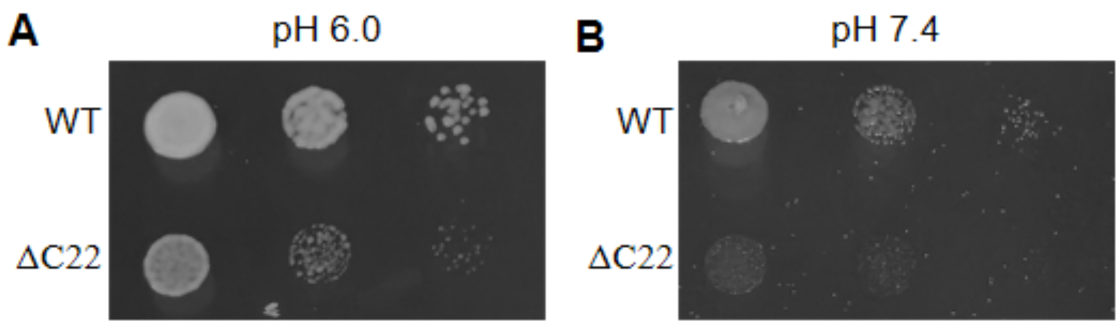

Supplementary Figure 12. Growth defects of yeast cells with truncated *pma1* (Pma1-ΔC22) at pH 6.0 and 7.4.

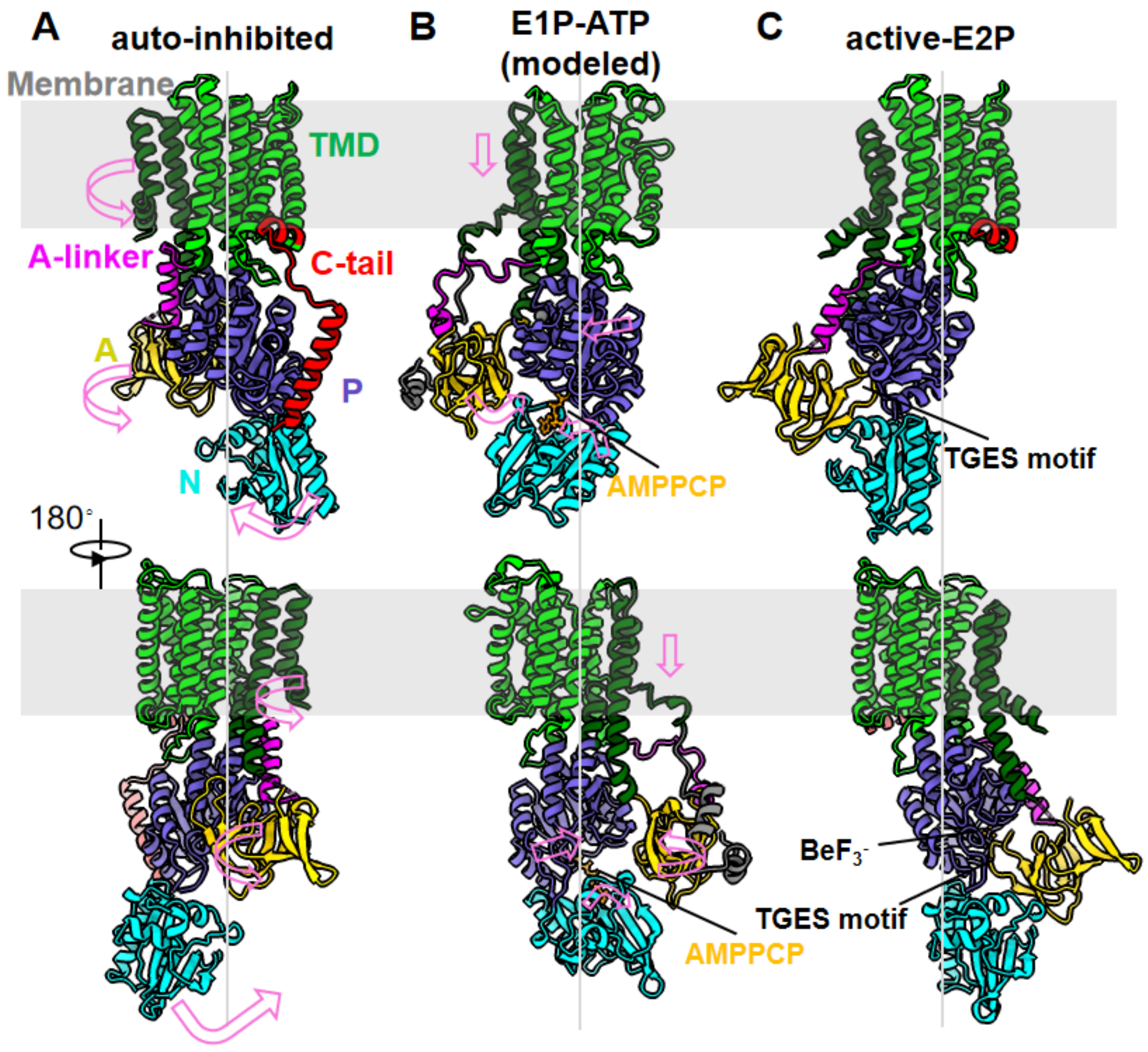

Supplementary Figure 13. Comparison of the Pma1 protomer at different stages of the functional cycle. (**A**) autoinhibited state. (**B**) E1-ATP state. Modeled with the plant H^+ ATPase AHA2 structure (PDB ID 5KSD). (**C**) Activated state. The grey line in the middle of each model marks the relative position of corresponding domains.

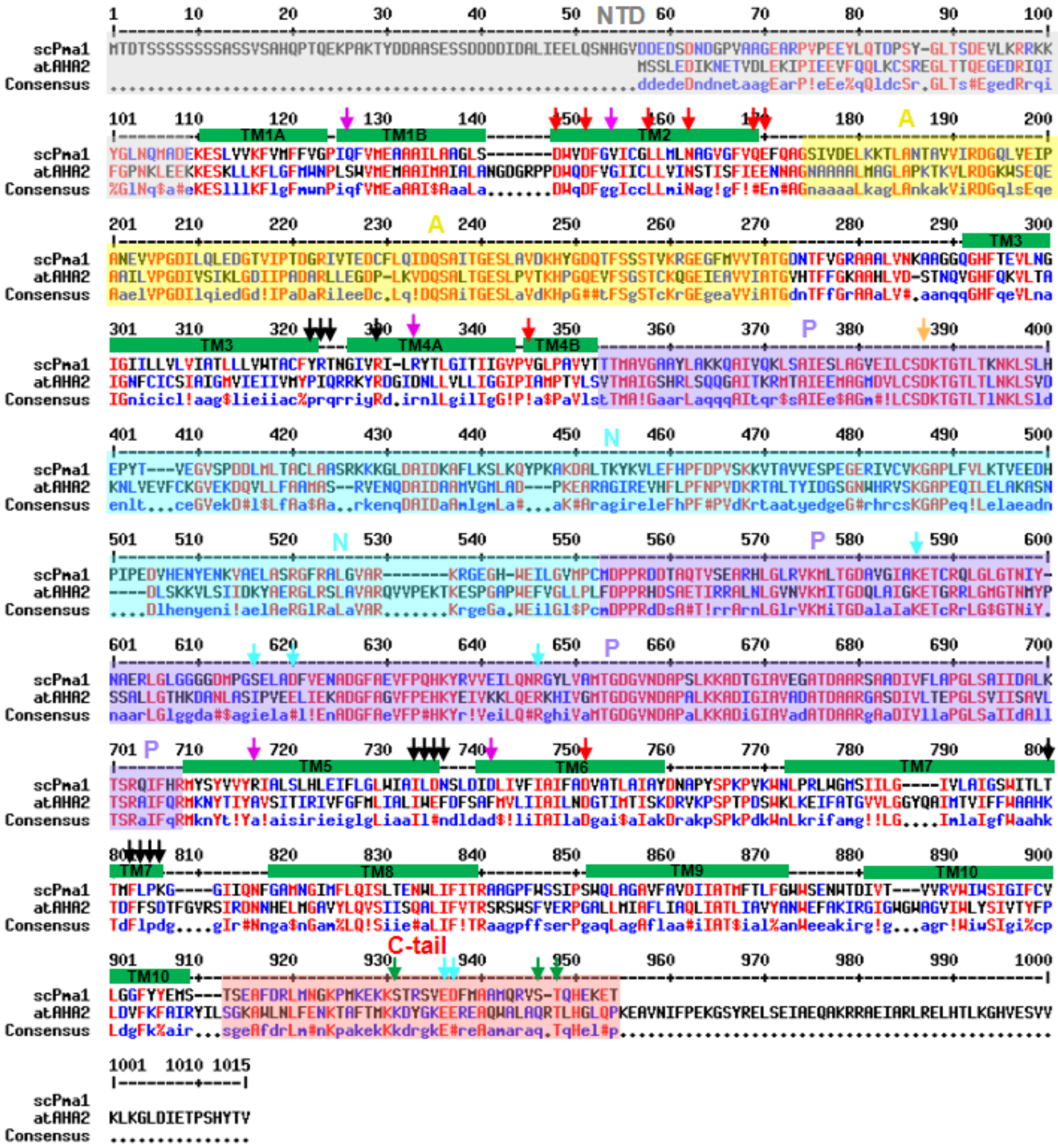

Supplementary Figure 14. Sequence alignment of yeast Pma1 with plant AHA2. Major domains and transmembrane helices are highlighted by different colors according to our autoinhibited Pma1 structure. Residues in the proton transporting path are highlighted by red (conserved) and purple (non-conserved) arrows. Three phosphorylation sites in the C-tail are highlighted by green arrows. Residues in the C-tail mediated interfaces are highlighted by cyan arrows. Residues in the exoplasmic subunit interface are highlighted by black arrows. Residues interacting with the lipids are highlighted by black arrows.

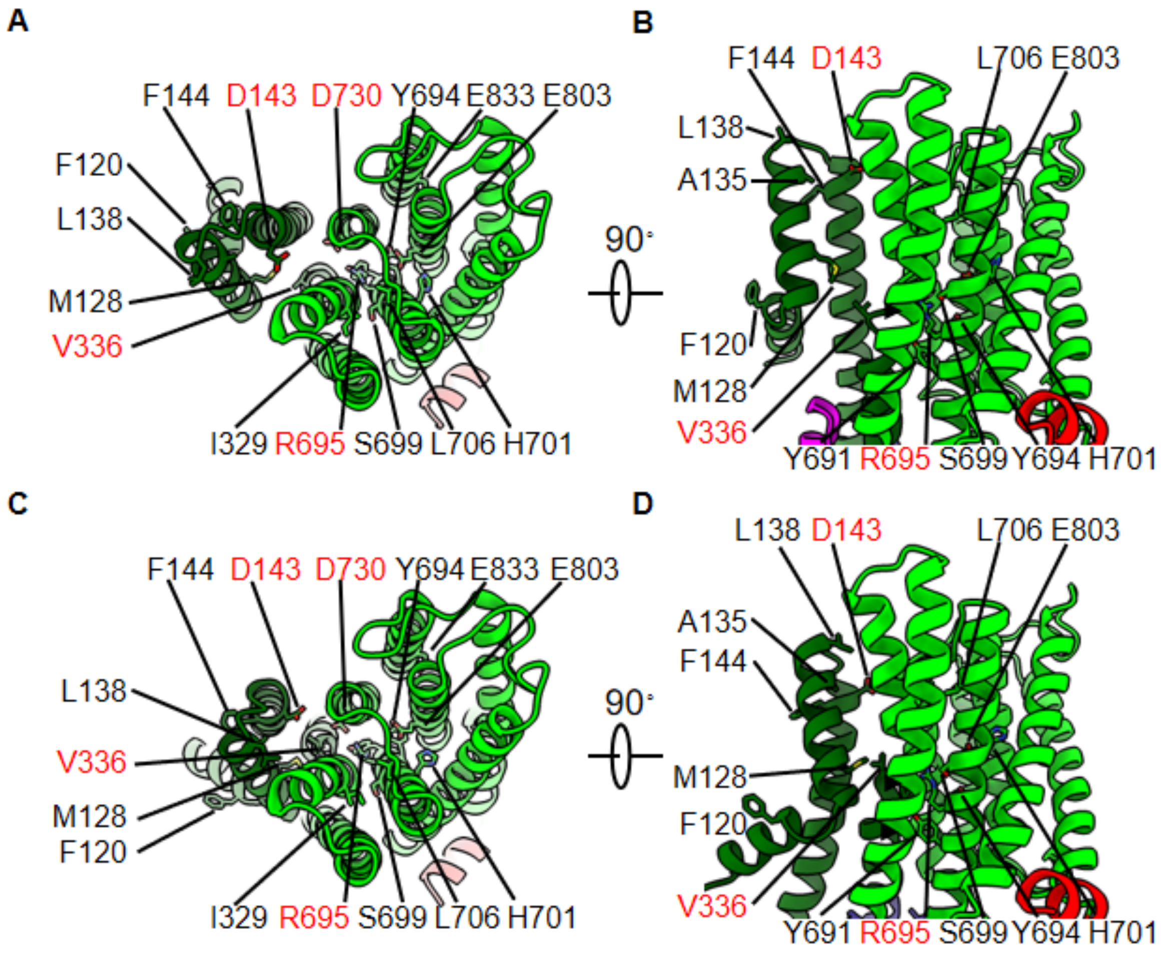

Supplementary Figure 15. Mapping of the selected previously reported mutations on the Pma1 structures. The selected mutations were either lethal or reduced the ATPase activity to <10% of the WT enzyme. The mutated residues are shown in sticks and mapped onto the Pma1 structures in the autoinhibited (**A-B**) and activated E2P state (**C-D**). Essential residues in the proposed substrate transporting path are highlighted in red.
