# Supplementary material for: Structure and activation mechanism of the hexameric plasma membrane H+-ATPase
Source: Nat Commun. 2021 Nov 8;12:6439. doi: 10.1038/s41467-021-26782-y (PMC8575881; doi:10.1038/s41467-021-26782-y)
Supplement: Supplementary file 2 — Description of Additional Supplementary Files [file 41467_2021_26782_MOESM2_ESM.pdf]

Description of Additional Supplementary Files

Supplementary Movie 1: The video morphs from autoinhibited Pma1 in pH7.4 to the activated Pma1 in pH6, shows the conformational changes and the details of the proton translocation path.
