# Supplementary material for: Structure and activation mechanism of the hexameric plasma membrane H+-ATPase
Source: Nat Commun. 2021 Nov 8;12:6439. doi: 10.1038/s41467-021-26782-y (PMC8575881; doi:10.1038/s41467-021-26782-y)
Supplement: Supplementary file 4 — Reporting summary [file 41467_2021_26782_MOESM4_ESM.pdf]

nature portfolio

Corresponding author(s): Lin Bai and Huilin Li

Last updated by author(s): Oct 16, 2021

Reporting Summary

Nature Portfolio wishes to improve the reproducibility of the work that we publish. This form provides structure for consistency and transparency in reporting. For further information on Nature Portfolio policies, see our Editorial Policies and the Editorial Policy Checklist.

Statistics

For all statistical analyses, confirm that the following items are present in the figure legend, table legend, main text, or Methods section.

n/a	Confirmed	
☐	☒	The exact sample size (n) for each experimental group/condition, given as a discrete number and unit of measurement
☐	☒	A statement on whether measurements were taken from distinct samples or whether the same sample was measured repeatedly
☒	☐	The statistical test(s) used AND whether they are one- or two-sided<br>*Only common tests should be described solely by name; describe more complex techniques in the Methods section.*
☒	☐	A description of all covariates tested
☒	☐	A description of any assumptions or corrections, such as tests of normality and adjustment for multiple comparisons
☐	☒	A full description of the statistical parameters including central tendency (e.g. means) or other basic estimates (e.g. regression coefficient) AND variation (e.g. standard deviation) or associated estimates of uncertainty (e.g. confidence intervals)
☒	☐	For null hypothesis testing, the test statistic (e.g. F, t, r) with confidence intervals, effect sizes, degrees of freedom and P value noted<br>*Give P values as exact values whenever suitable.*
☒	☐	For Bayesian analysis, information on the choice of priors and Markov chain Monte Carlo settings
☒	☐	For hierarchical and complex designs, identification of the appropriate level for tests and full reporting of outcomes
☒	☐	Estimates of effect sizes (e.g. Cohen's d, Pearson's r), indicating how they were calculated

Our web collection on statistics for biologists contains articles on many of the points above.

Software and code

Policy information about availability of computer code

Data collection	Cryo-EM data collection used SerialEM(version 3.8.8) in Titan Krios and EPU as implemented in Arctica by the manufacturer Thermo-Fisher Scientific.
Data analysis	RELION-3.1, MotionCorr2-1.4.O, CTFFIND4.1, cryoSPARC-3.1, ChimeraX 1.2.5, Pymol (2.06), Coot (version 0.9.5), Phenix (version 1.19.2-4158-000), and MolProbity (version 4.5).

For manuscripts utilizing custom algorithms or software that are central to the research but not yet described in published literature, software must be made available to editors and reviewers. We strongly encourage code deposition in a community repository (e.g. GitHub). See the Nature Portfolio guidelines for submitting code & software for further information.

Data

Policy information about availability of data

All manuscripts must include a data availability statement. This statement should provide the following information, where applicable:

- Accession codes, unique identifiers, or web links for publicly available datasets
- A description of any restrictions on data availability
- For clinical datasets or third party data, please ensure that the statement adheres to our policy

The cryo-EM 3D maps and the corresponding atomic models of the yeast Pma1 hexamer have been deposited at the EMDB database and the RCSB PDB with the respective accession codes of EMD-31986 and 7VH5 (Pma1-pH7-C1) (https://doi.org/10.2210/pdb7vh5/pdb), EMD-31987 (Pma1-pH7-C6), EMD-31988 and 7VH6 (Pma1-pH6-BeF-Conf1-C1) (https://doi.org/10.2210/pdb7vh6/pdb), EMD-31989 (Pma1-pH6-BeF-Conf1-C6), EMD-31990 (Pma1-pH6-BeF-Conf2), EMD-31991 (Pma1-pH6-C1), EMD-31992 (Pma1-pH6-highsalt), and EMD-31993 (Pma1-pH7-BeF).

Field-specific reporting

Please select the one below that is the best fit for your research. If you are not sure, read the appropriate sections before making your selection.

- [x] Life sciences
- [ ] Behavioural & social sciences
- [ ] Ecological, evolutionary & environmental sciences

For a reference copy of the document with all sections, see nature.com/documents/nr-reporting-summary-flat.pdf

Life sciences study design

All studies must disclose on these points even when the disclosure is negative.

Sample size	We collected five cryo-EM datasets: 1) 4152 micrographs with Pma1 at pH7.4; 2) 2910 micrographs with Pma1 bound to BeFx at pH7.4; 3) 3436 micrographs with Pma1 in buffer at pH6.0; 4) 3492 micrographs with Pma1 bound to BeFx in buffer at pH6.0; 5) 242 micrographs with Pma1 in buffer containing 500 mM NaCl at pH6.0. The sample size was not predetermined. The sample size was deemed appropriate when it led to 3D maps at desired resolution.
Data exclusions	"Bad" raw particles that did not produce 2D class averages or 3D class maps with defined features were excluded after 2D and 3D classifications. This criteria is empirical but is a standard image processing practice in the cryoEM community.
Replication	Reproducibility resides in the large number of particles used to derive at the final 3D maps or 2D averages. The reliability and the resolution is measured by gold-standard Fourier shell correlation. Replication efforts with multiple refinement runs (n>3) yielded was successful, yielding similar 3D maps.
Randomization	The raw particles were automatically selected by computer program (RELIONS 3.0). Randomization is not relevant to this study.
Blinding	The investigators were not blinded to the specific data points during data collection and analysis. There is no need for blinding in this type of study.

Reporting for specific materials, systems and methods

We require information from authors about some types of materials, experimental systems and methods used in many studies. Here, indicate whether each material, system or method listed is relevant to your study. If you are not sure if a list item applies to your research, read the appropriate section before selecting a response.

Materials & experimental systems

n/a	Involved in the study	
[x]	[ ]	Antibodies
[ ]	[x]	Eukaryotic cell lines
[x]	[ ]	Palaeontology and archaeology
[x]	[ ]	Animals and other organisms
[x]	[ ]	Human research participants
[x]	[ ]	Clinical data
[x]	[ ]	Dual use research of concern

Methods

n/a	Involved in the study	
[x]	[ ]	ChIP-seq
[x]	[ ]	Flow cytometry
[x]	[ ]	MRI-based neuroimaging

Eukaryotic cell lines

Policy information about cell lines

Cell line source(s)	yeast strain BY4742
Authentication	Purchased from Horizon Discovery
Mycoplasma contamination	The cell line was not tested for mycoplasma contamination
Commonly misidentified lines (See ICLAC register)	n/a
